# Supplementary material for: Water-mediated ion transport in an anion exchange membrane
Source: Nat Commun. 2025 Jan 28;16:1099. doi: 10.1038/s41467-024-55621-z (PMC11775218; doi:10.1038/s41467-024-55621-z)
Supplement: Supplementary file 1 — Supplementary Information [file 41467_2024_55621_MOESM1_ESM.pdf]

Supplementary Information

Water-Mediated Ion Transport in an Anion Exchange Membrane

Zhongyang Wang,[1,2,†] Ge Sun,[1,3,†] Nicholas H. C. Lewis,[4] Mrinmay Mandal,[5] Abhishek Sharma,[1,6] Mincheol Kim,[1] Joan M. Montes de Oca,[1] Kai Wang,[1] Aaron Taggart,[7] Alex B. Martinson,[7] Paul A. Kohl,[5] Andrei Tokmakoff,[4,*] Shrayesh N. Patel,[1,*] Paul F. Nealey [1,7,*] and Juan J. de Pablo [1,3,7,*]

[1]Pritzker School of Molecular Engineering, University of Chicago, Chicago, Illinois 60637, USA

[2]Department of Chemical and Biological Engineering, The University of Alabama, Tuscaloosa, Alabama 35487, USA

[3]Department of Chemical and Biomolecular Engineering, Tandon School of Engineering, New York University, Brooklyn, New York 11201, USA, and Courant Department of Computer Science, and Department of Physics, New York University, New York 10012, USA

[4]Department of Chemistry, James Franck Institute, The University of Chicago, Chicago, Illinois 60637, USA

[5]School of Chemical and Biomolecular Engineering, Georgia Institute of Technology, Atlanta, Georgia 30332, USA

[6]Department of Chemical Engineering, The Cooper Union for the Advancement of Science and Art, New York, New York 10003, USA

[7]Materials Science Division, Argonne National Laboratory, Lemont, Illinois 60439, USA

Corresponding author*: tokmakoff@uchicago.edu; shrayesh@uchicago.edu; nealey@uchicago.edu; jjd8110@nyu.edu

[†]These authors contributed equally to this work

Supplementary Section 1: Conventional wisdom about water absorption and ion transport in an anion exchange membrane

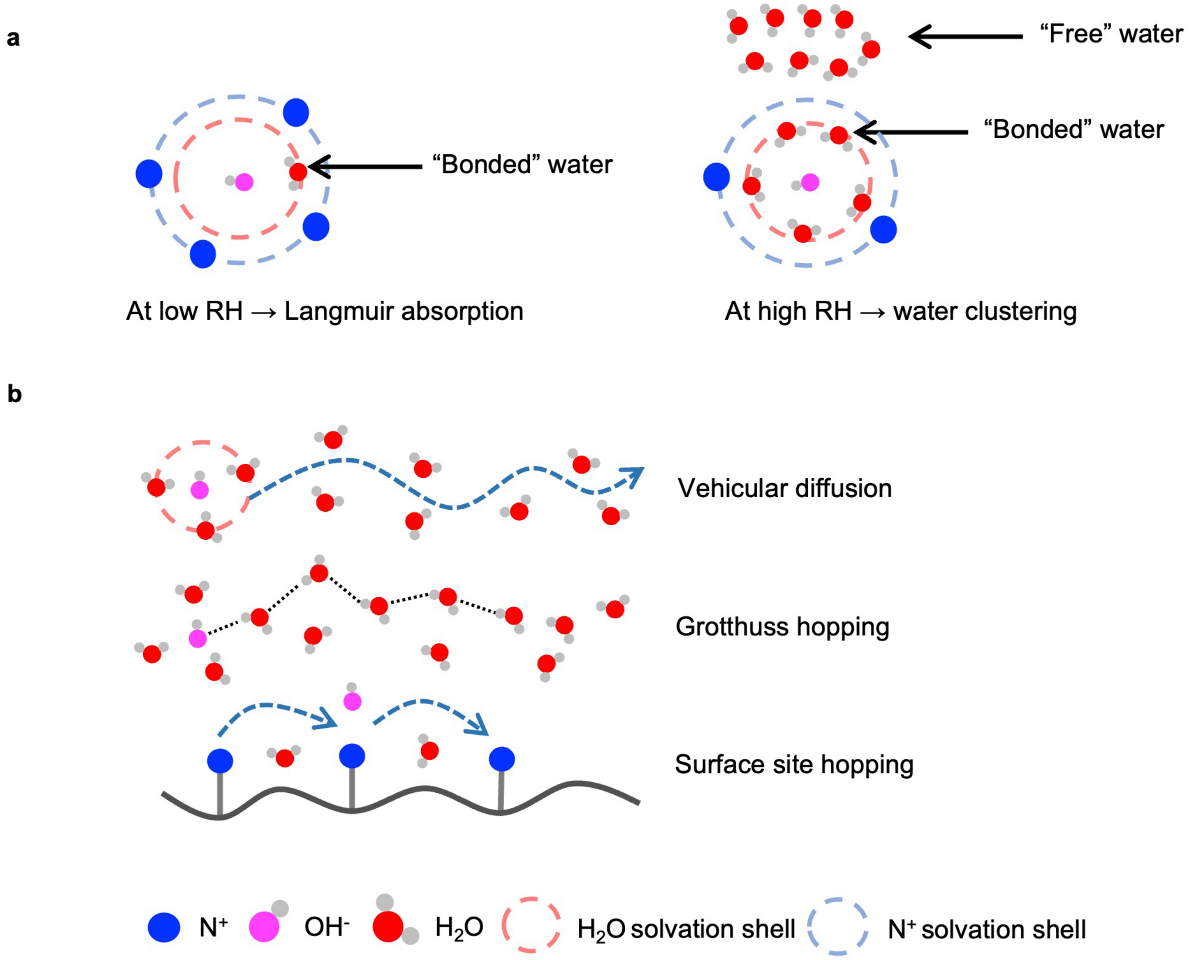

Supplementary Fig. 1 | Water absorption and ion transport. **a**, Illustration of different water states in an AEM. **b**, Illustration of ion transport mechanisms in an AEM.

Anion transport in an AEM is governed by three transport mechanisms: surface site hopping, vehicular, and Grotthuss (for OH^- transport) [1–3]. The schematic of transport mechanisms is shown in Supplementary Fig. 1b. At low hydration levels, anion transport is dominated by hopping of anions between solvation sites composed of cationic groups (e.g., trimethylammonium, trimethylphosphonium, and trimethylsulfonium)[4]. This surface site hopping mechanism is correlated to the segmental mobility and solvation environment of the polymer chain[5–8]. At higher

water content, vehicular and Grotthuss mechanisms begin to govern the overall anion transport. Vehicular transport occurs when water molecules fully dissociate anions from cationic groups and the anions diffuse with a largely intact solvation shell. The Grotthuss transfer mechanism, where OH^- (or H^+) is understood as a defect in the hydrogen-bonded water network that moves largely via the transfer of protons[9–11], facilitates the anomalously high diffusion for these species in aqueous environments.

Supplementary Section 2: Experimental methods and Material characterizations

S2.1 Synthesis of PBBNB$^+$Br$^-$ anion exchange membranes

Synthesis method of poly(bromo butylnorbornene) (PBBNB) from bromo butyl norbornene (BBNB) can be found in the experimental section and our previous report[17]. We synthesized BBNB via Diels-Alder reaction and polymerize it to PBBNB via vinylic addition polymerization method and the obtained polymer has a molecular weight (M_n) of 68.356 kg/mol with dispersity ($Đ$) of 1.20. The synthesis method can be found in the experimental section and characterizations including ^{1}H-NMR and size exclusion chromatography (SEC) measurements are shown in Supplementary Fig. 2&3. A vapor infiltration reaction (VIR) was used to functionalize neutral PBBNB thin film (*ca.* 65 nm) to charged poly(butylnorbornene) (PBBNB$^+$Br$^-$), as shown in Supplementary Fig. 4. FTIR was used to monitor the reaction extent of the VIR and the PBBNB$^+$Br$^-$ film reached a maximum IEC of 3.20 mmol/g at vapor infiltration time (t_{VIR}) of 5 hours. Detailed analysis and discussions can be found in Supplementary Discussion and Supplementary Fig. 5&6. After the VIR, optical microscopic images were taken for PBBNB$^+$Br$^-$ thin films before and after reacting with trimethylamine (TMA) to confirm that there was no dewetting phenomenon observed (see Supplementary Fig. 7). To calculate ion conductivities and

understand ion transport in PBBNB$^+$Br$^-$, water uptakes and film thickness expansion were measured as a function of RH, as shown in Supplementary Figs. 8-14. Water uptakes of PBBNB$^+$Br$^-$ was fitted with Park model and water volume fraction (ϕ_w) was calculated based on the thickness expansion of the film. Calculations can be found in Supplementary Discussion and Supplementary Figs. 15-17. Note: error bars are standard error obtained with n = 3 samples.

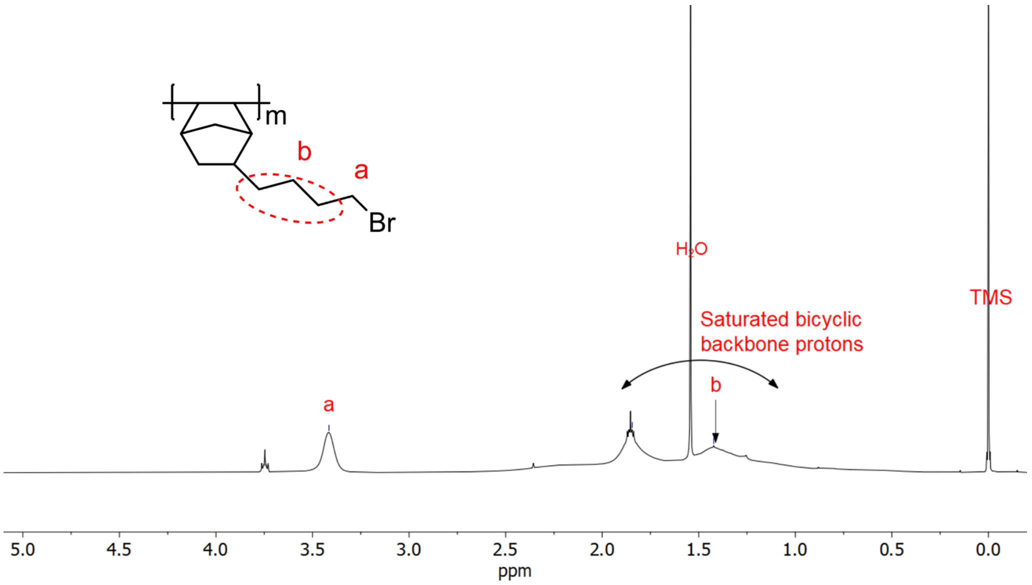

Supplementary Fig. 2 | ^{1}H-NMR spectra of PBBNB. The peak "b" corresponds to the protons on alkyl side chain and peak "a" corresponds to the protons from methyl group that connects to the bromide atom. The 1H NMR was done in deuterated chloroform with TMS. TMS was used as internal standard for calibrating the chemical shift for ^{1}H-NMR.

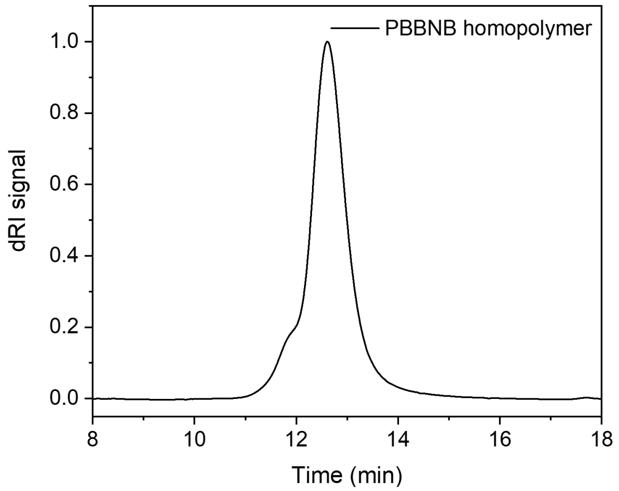

Supplementary Fig. 3 | SEC trace of PBBNB. The M_n of PBBNB is 68.356 kDa with *Đ* of 1.20. The theoretical IEC of PBBNB is 3.48 mmol/g.

S2.2 Fabrication of PBBNB$^+$Br$^-$ thin films

To convert neutral PBBNB to charged PBBNB$^+$Br$^-$, a vapor infiltration reaction (VIR) was conducted. The chemical reaction scheme is shown in Supplementary Fig. 4a. The schematic illustration of preparing PBBNB$^+$Br$^-$ anion exchange thin films via VIRs is shown in Supplementary Fig. 4b. PBBNB was first dissolved in chloroform and the solution was spin-casted on top of different substrates for various purposes. The samples were then thermally annealed to evaporate any residual solvent. The VIR was conducted by placing a thin film sample adjacent to a vial that contains 4 mL 45 wt% TMA water solution.

The aim of functionalizing PBBNB thin films via VIRs is to maximize the fraction of quaternary ammonium group while minimizing the exposure time to TMA solution to prevent any dewetting of the polymer thin film. The fraction of quaternary ammonium at different vapor exposure time was monitored by FTIR. Supplementary Fig. 5a shows a series of FTIR spectra ranging from 500 to 1550 cm^{-1} of the polymer thin films at different VIR times. For the neat

PBBNB thin film (VIR time, $t_{VIR} = 0$), C-H stretching from polymer backbone and alkyl side chain is at *ca.* 1489 cm^{-1}, and C-Br stretching from the end group of side chain is at *ca.* 648 $cm^{-1}$and 563 cm^{-1}. As TMA reacts with C-Br end groups via the nucleophilic substitution reaction, C-N^+ stretching peaks appear at *ca.* 1489 cm^{-1}, 970 cm^{-1} and 908 cm^{-1}. Red dash line is added as a guide to the eye to highlight the appearance of the C-N^+ stretching mode, and purple circle around 648 cm^{-1} and 563 cm^{-1} highlights the disappearance of C-Br stretching mode. Supplementary Fig. 5b presents the same series of FTIR spectra ranging from 2400 to 3800 cm^{-1}. As more C-Br is converted to C-N^+ from the reaction, the polymer thin films become hygroscopic. In turn, water molecules are bounded with the cationic groups and O-H stretching from water molecules is observed at *ca.* 3440 cm^{-1}. A blue dash line is added to highlight the appearance of O-H stretching mode.

To approximate the reaction extent as a function of exposure time, fraction of quaternary ammonium group is defined by equation (1). The fraction of quaternary ammonium group reaches 92% at $t_{VIR} = 5$ h and remains unchanged upon further exposed to TMA solution (see Supplementary Fig. 6a). All samples were exposed to TMA solution for 5 h and $t_{VIR} = 5$ h is viewed as the saturation point of the VIR. Supplementary Fig. 6b shows ion exchange capacities (IECs) of $PBBNB^+Br^-$ films as a function of t_{VIR} calculated from faction of quaternary ammonium groups. At $t_{VIR} = 5$ h, the calculated IEC of the $PBBNB^+Br^-$ film is 3.20 mmol/g. The theoretical IEC of the $PBBNB^+Br^-$ film is calculated as 3.48 mmol/g by equation (2).

$$\text{Fraction of quaternary ammonium group} = \frac{\text{Peak area of C}-\text{N}^{+}\text{ stretching}}{\text{Peak area of C}-\text{N}^{+}\text{ stretching}+\text{Peak area of C}-\text{Br stretching}} \times 100\% \quad (1)$$

$$\text{Theoretical IEC } = \frac{\text{Number of repeating unit}}{\text{Polymer molecular weight}} \times 1000, \text{mmol/g}$$

$$\text{Experimental IEC} = \text{Theoretical IEC} \times \text{fraction of quaternary ammonium group, mmol/g} \quad (2)$$

The thickness of neutral PBBNB film is *ca.* 65 nm and the obtained fully functionalized $PBBNB^+Br^-$ anion exchange film is *ca.* 98 nm at ambient environment, as confirmed by ellipsometry. In our previous report, we have reacted poly(styrene-block-2-vinylpyridine) (PS-b-P2VP) block copolymer with methyl iodide to form PS-b-P2VP-based anion exchange thin films via VIRs [7]. The methyl iodide molecule was able to infiltrate the whole PS-b-P2VP film and reach reasonable homogeneity, as confirmed by FTIR and XPS. Optical microscopic images were taken for $PBBNB^+Br^-$ thin films before and after reacting with TMA to confirm that there was no dewetting phenomenon observed (see Supplementary Fig. 7)

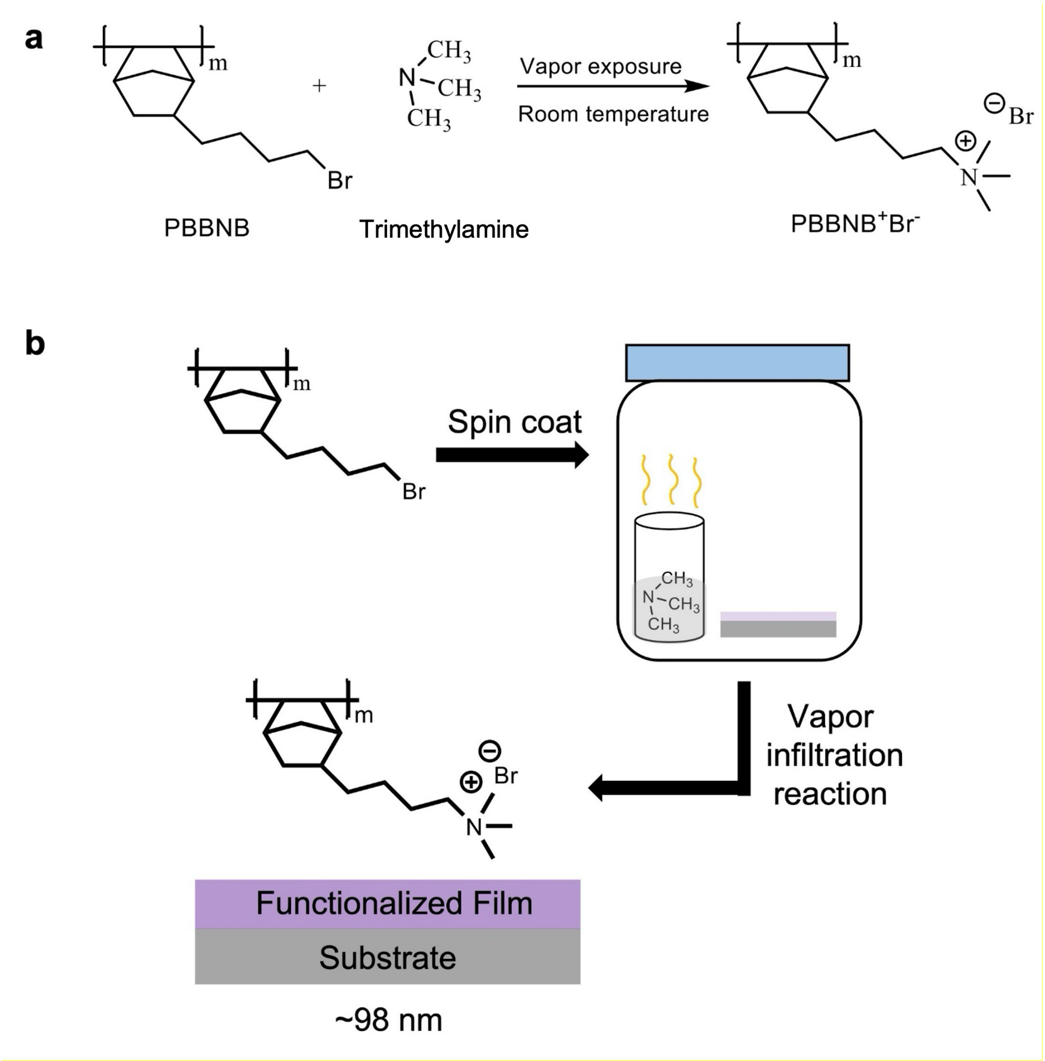

Supplementary Fig. 4 | Process scheme to functionalize polynorbornene films. a, Chemical reaction of brominated polynorbornene with trimethylamine to synthesize $PBBNB^{+}Br^{-}$. **b,** Schematic illustration for the preparation of $PBBNB^{+}Br^{-}$ thin films via vapor infiltration reactions.

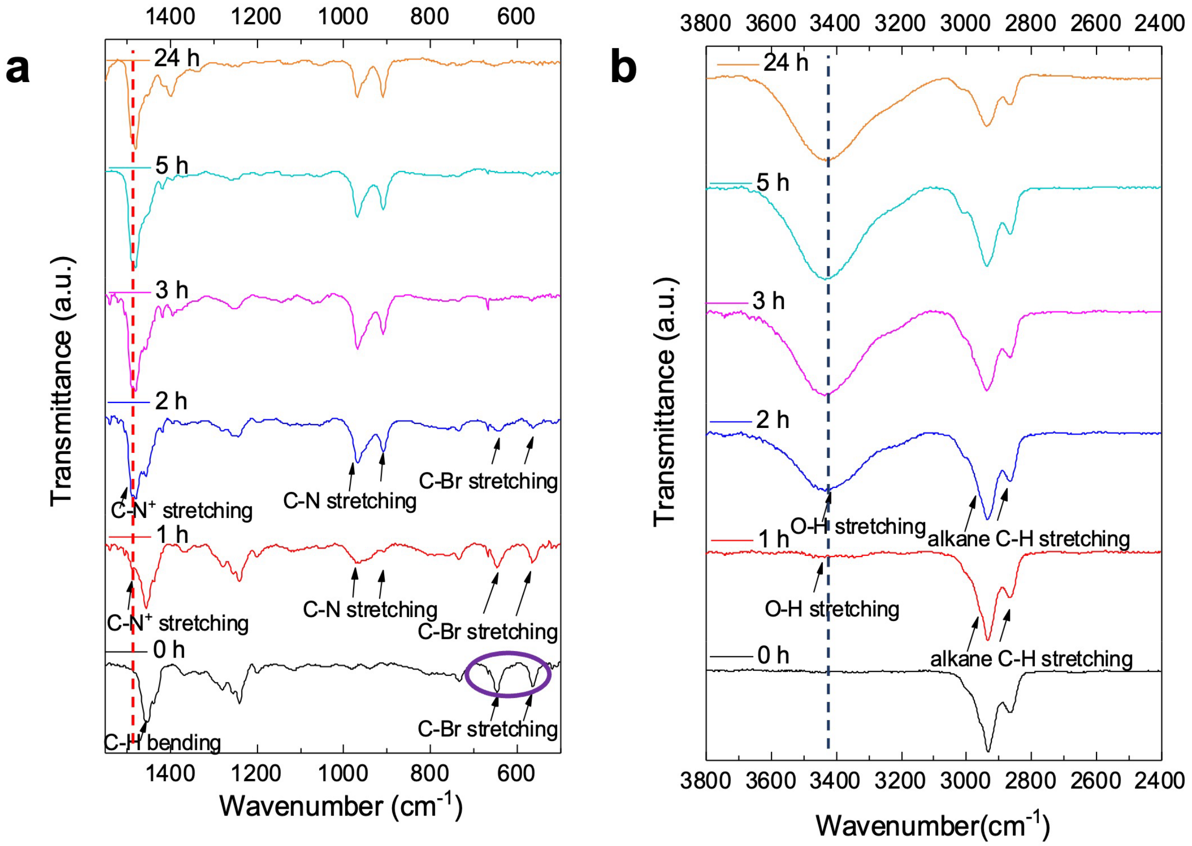

Supplementary Fig. 5 | FTIR spectrum of the AEM as a function of reaction time. a, FTIR spectrum of PBBNB$^+$Br$^-$ anion exchange thin films in the range of 500 to 1550 cm^{-1}. The spectrum was measured as a function of vapor exposure time. The red dash line indicates the appearance of C-N$^+$ stretching from the reaction. The purple circle indicates the existing C-Br stretching from neutral polynorbornene. **b,** FTIR spectrum of PBBNB$^+$Br$^-$ anion exchange thin films in the range of 2400 to 3800 cm^{-1}. The spectrum was measured as a function of vapor exposure time. The blue dash line indicates the appearance of O-H stretching from the absorbed water molecules by quaternary ammonium.

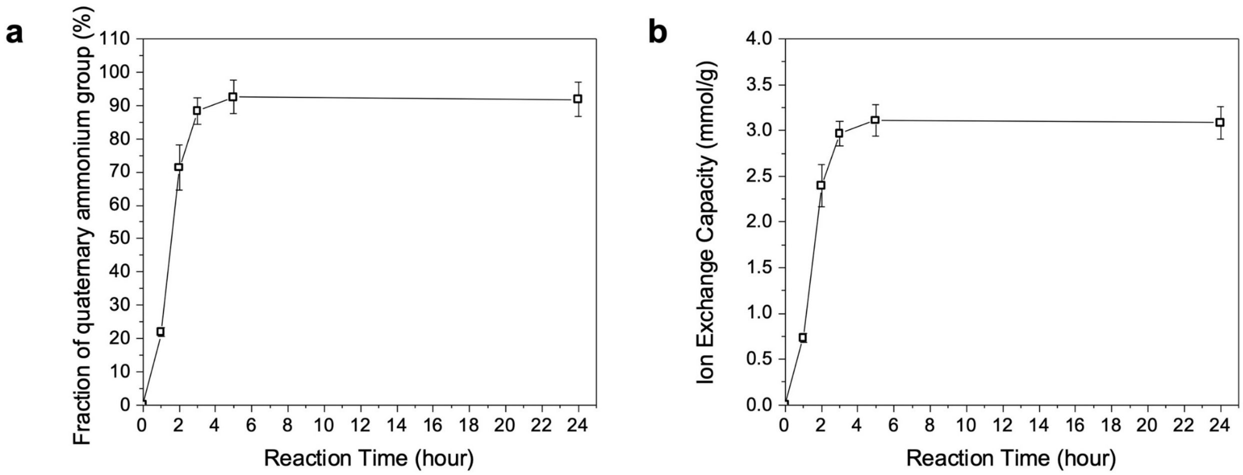

Supplementary Fig. 6 | Change of ion exchange capacity (IEC) of the AEM as a function of reaction time. a, Fraction of quaternary ammonium group in the $PBBNB^{+}Br^{-}$ anion exchange thin films as a function of vapor exposure time. **b**, IEC as a function of reaction time. After 5 hours reaction, the film reaches its highest IEC, 3.20 mmol/g. The conversion rate of vapor infiltration reaction is 92%. Note: error bars are standard error obtained with $n = 3$ samples.

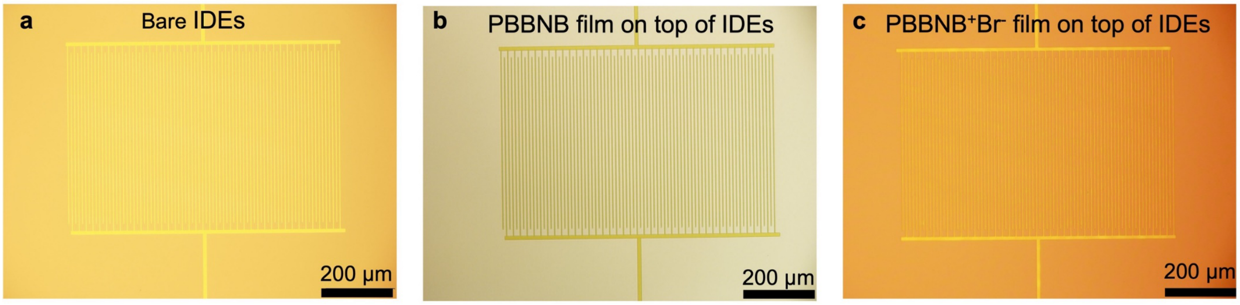

Supplementary Fig. 7 | Optical images of interdigitated electrode arrays (IDEs). a, Optical microscopic images of bare IDEs. **b**, neutral polynorbornene (PBBNB) and **c**, charged polynorbornene-based anion exchange thin film ($PBBNB^{+}Br^{-}$) on top of IDEs. There was no de-wetting phenomenon observed during the film deposition processes and vapor infiltration reactions.

S2.3 Water uptakes, and film thickness expansion of $PBBNB^+Br^-$

To investigate solvation environments for Br^- ion at different hydration levels, water uptakes of $PBBNB^+Br^-$ anion exchange materials were measured by dynamic vapor sorption (DVS). The water uptake values are averaged based on the sorption and the desorption process (shown in Supplementary Fig. 8). The hydration number (λ) (Supplementary Table 1) was calculated based on water uptakes of $PBBNB^+Br^-$ at different RH and was further used in MD simulations to determine the number of water molecules in each system. The water uptake of $PBBNB^+Br^-$ first increases slowly from 0% RH to 15% RH and increases linearly from 15 % RH to 65 % RH. Above 65 % RH, water uptake increases rapidly to 95% RH. The water uptake data can be fitted to the Park model[15], which is governed by Langmuir absorption at low RH, Henry absorption at median RH, and clustering at high RH. At low RH, water molecules will be accumulated on the surface of the polymer film, and the formation of a monolayer can be described by Langmuir model. At median RH, an ordinary dissolution mechanism described by the Henry model dominates the absorption of water molecules. At high RH, water molecules show bulk water clustering behavior, and the absorption can be described by the clustering model. The fitting results can be seen in Supplementary Fig. 9. The water uptakes of $PBBNB^+Br^-$ measured at 35 °C and 40 °C are shown in Supplementary Fig. 10.

To calculate ionic conductivities of $PBBNB^+Br^-$ thin films at different RH values, an *in situ* spectroscopic ellipsometer was built to measure film thicknesses. The experimental setup is shown in Supplementary Fig. 11, and a detailed description of the measurements can be found in the experimental section. The film thickness was measured from 25% RH up to 90% RH as shown in Supplementary Fig. 12. The film thickness is 98.2 nm at 25% RH and increases linearly to 111.1 nm at 65% RH (see linear fit in Supplementary Fig. 13). Above 65% RH, the thickness increases

rapidly (see polynomial fit in Supplementary Fig. 14). Since the film thickness at 95% can not be acquired due to the limitation of RH generator, a polynomial equation is used to extrapolate the thickness at 95% RH. Voth and co-workers simulated ion transport in a poly (vinyl benzyltrimethylammonium) (PVBTMA) system. They investigated the density of the polymer as a function of hydration number λ. The density of the polymer increases with the increase of λ up to λ = 7 and then declines [11]. As λ increases, water molecules will first fill up the cavities in the polymer at low and median hydration levels and the volume will not change dramatically, which likely causes the linear increase of the film thickness. After filling up the cavities in the polymer, the addition of water molecules will dramatically increase the polymer volume, which is likely the reason of the rapid increase of the film thickness at high hydration levels. Interestingly, water uptakes (Supplementary Fig. 8) and thickness expansion (Supplementary Fig. 12) of $PBBNB^+Br^-$ show very similar behaviors at median RH (25% RH to 65% RH) and high RH (65% RH to 95% RH). To understand the addition of water molecules in expanding the thin film, thickness expansion of $PBBNB^+Br^-$ was plotted as a function of water uptakes and the figure shows a linear relationship with a slope of 1.083 (see Supplementary Fig. 15). The linear relationship indicates that the thickness expansion of the film is attributed to the added volume of water molecules.

Water volume fraction (ϕ_w) was calculated by a simplified method based on the change of the film thickness (see Supplementary Fig. 16) and plotted as a function of RH (Supplementary Fig. 17). Since we show our conductivity and simulation results as a function of RH later in the paper, one can refer Supplementary Fig. 17 to know the corresponding ϕ_w. Due to the temperature limitation of the DVS instrument (Intrinsic), water uptakes of $PBBNB^+Br^-$ were measured from 25 °C to 40 °C and there were no significant changes observed (Supplementary Fig. 10). Elabd and co-workers measured water uptakes of poly(ionic liquids) (PILs)-based AEMs from 30 °C to

60 °C at 90% RH and water uptakes remain constant.[32] Water uptake measurements of PBBNB$^+$Br$^-$ agree with the measurements of PIL-based AEMs.

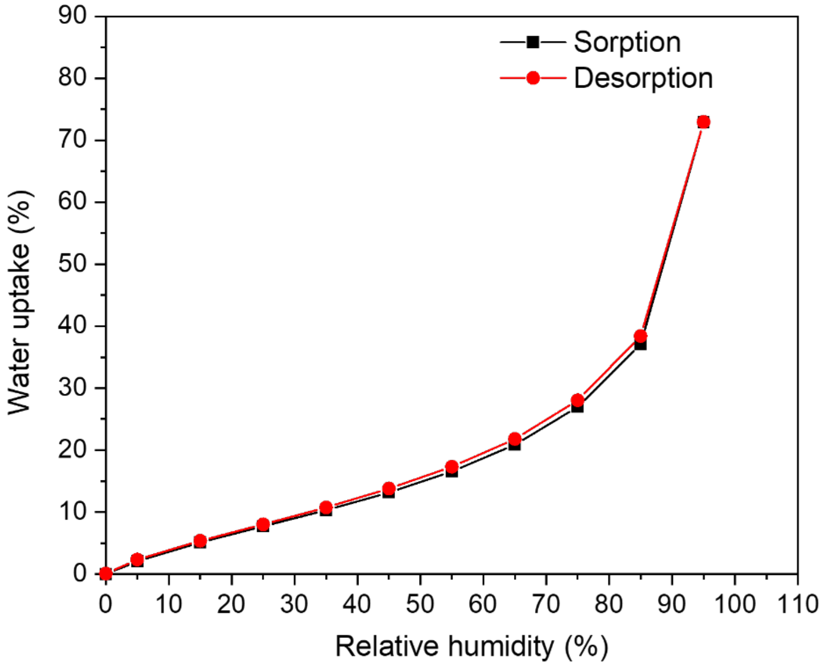

Supplementary Fig. 8 | Water uptake values of PBBNB$^+$Br$^-$ anion exchange materials measured by dynamic vapor sorption.

Supplementary Table 1. Hydration number (λ) of PBBNB$^+$Br$^-$ anion exchange materials are calculated by the following equation (3).

$$\lambda = \frac{\frac{mH_2O}{18.02g/mol}}{nN^+} \quad (3)$$

Relative humidity (%)	Hydration number (λ)
0	0.0083
5	0.39
15	0.94
25	1.41
35	1.88
45	2.41
55	3.03
65	3.82
75	4.92
85	6.76
95	13.06

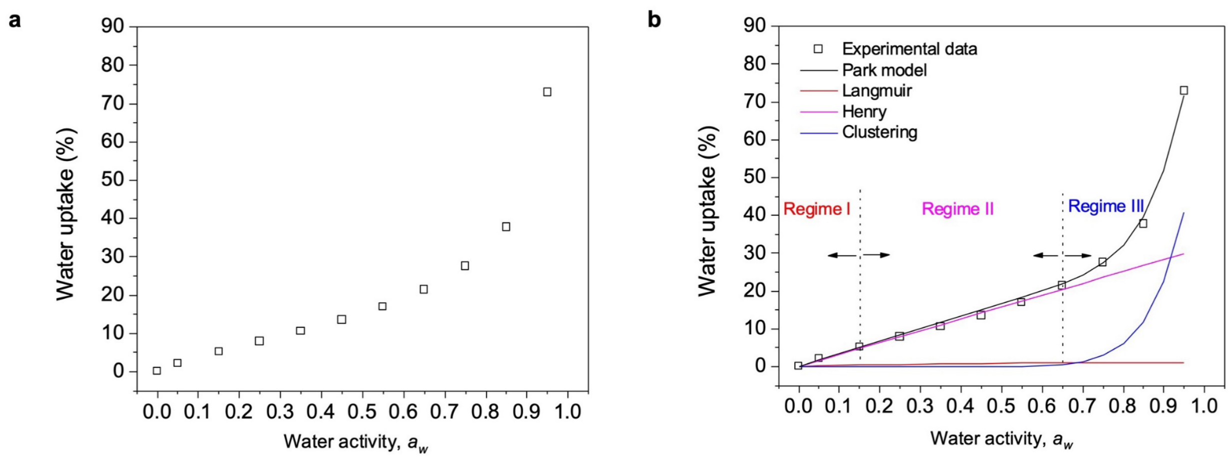

Supplementary Fig. 9 | Water absorption behaviors of PBBNB$^+$Br$^-$. a, Water uptake of PBBNB$^+$Br$^-$ as a function of water activity (a_w). **b,** Water absorption of PBBNB$^+$Br$^-$ was fitted by the Park model[12–14] and the absorption behaviors are governed by Langmuir absorption (Regime I) at low water activity, Henry absorption at median water activity, and clustering behavior at high water activity. Park equation (4) and fitting parameters are shown below.

Water uptake = $\frac{a_L K_L a_w}{1+K_L a_w} + K_H a_w + nK_A {a_w}^n$ (4)

where a_L is the specific site capacity, K_L is an affinity constant, K_H is the Henry's law coefficient, K_A is the aggregation equilibrium constant, and n is the size of the aggregate.

To fit experimental data with Park model, we first fitted the data in Regime II to calculate K_H. Then, we fixed K_H and fitted data in Regime I and Regime III to obtain other parameters from Langmuir and clustering contributions. The fitting results are shown below.

$$a_L = 1.43$$

$$K_L = 2.95$$

$$K_H = 31.46$$

$$K_A = 6.46$$

$$n = 11.14$$

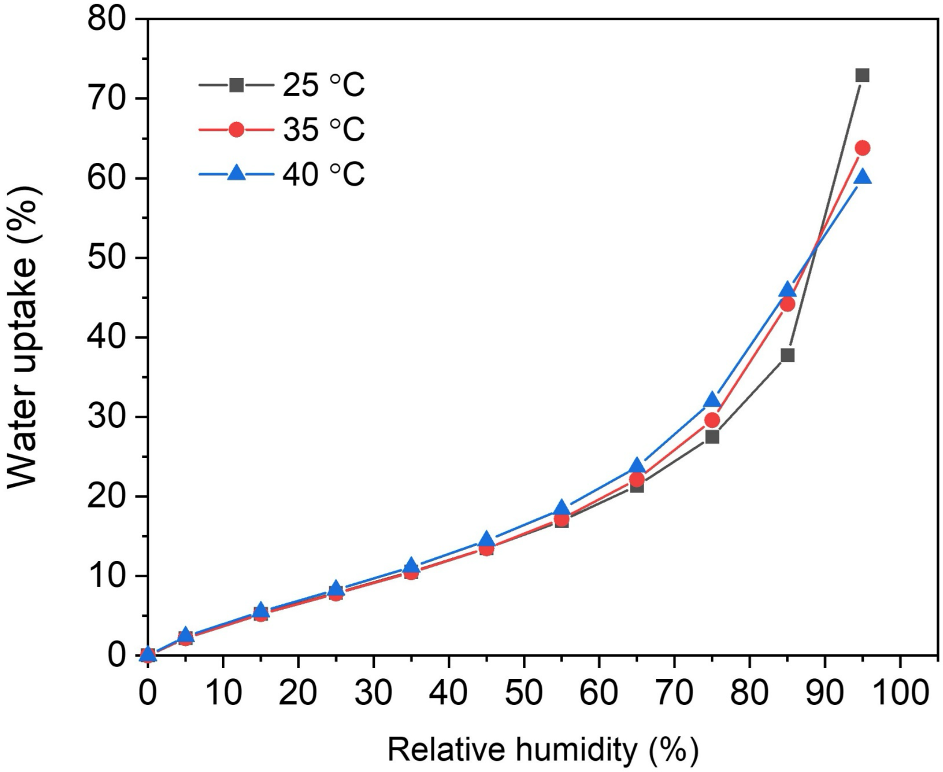

Supplementary Fig. 10 │ Water uptake of PBBNB$^+$Br$^-$ as a function of RH and temperature. Due to the limitation of DVS Intrinsic plus instrument, the samples can only be heated up to 40 °C. The water uptakes of the AEM remains relatively constant from 25 °C to 40 °C.

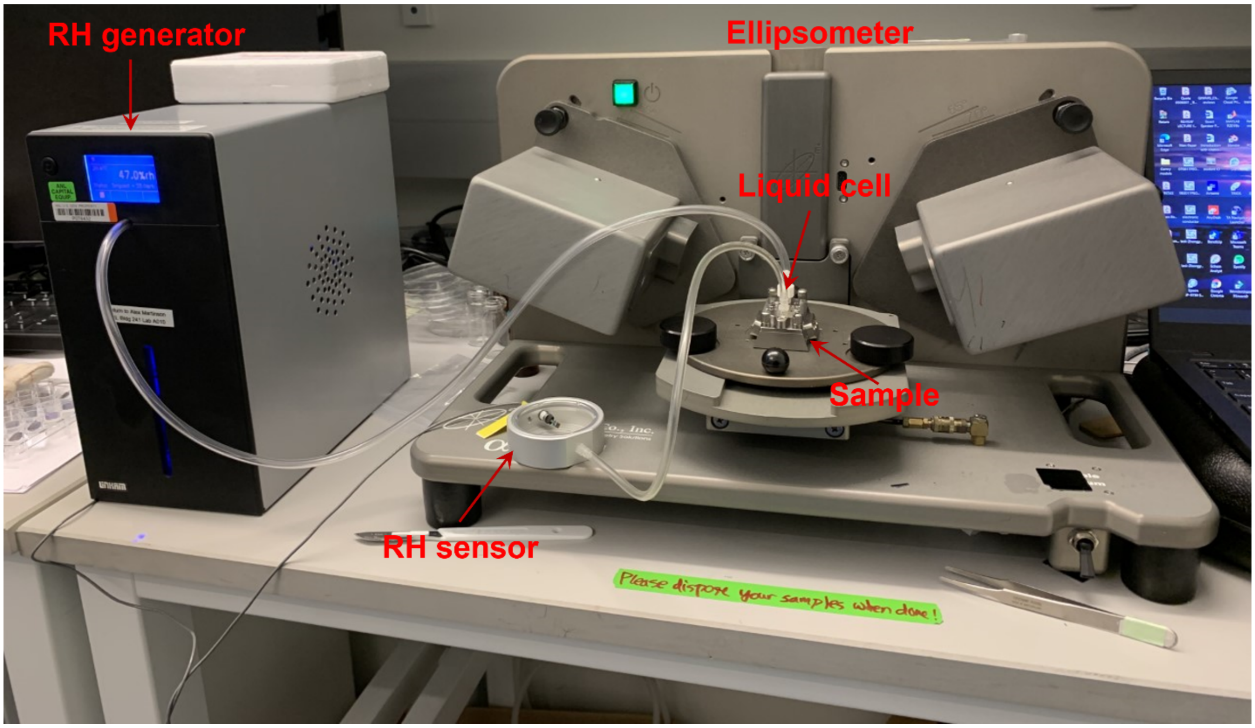

Supplementary Fig. 11 | Picture of an *in situ* ellipsometer used to measure PBBNB^{+}Br^{-} anion exchange thin films as a function of RH.

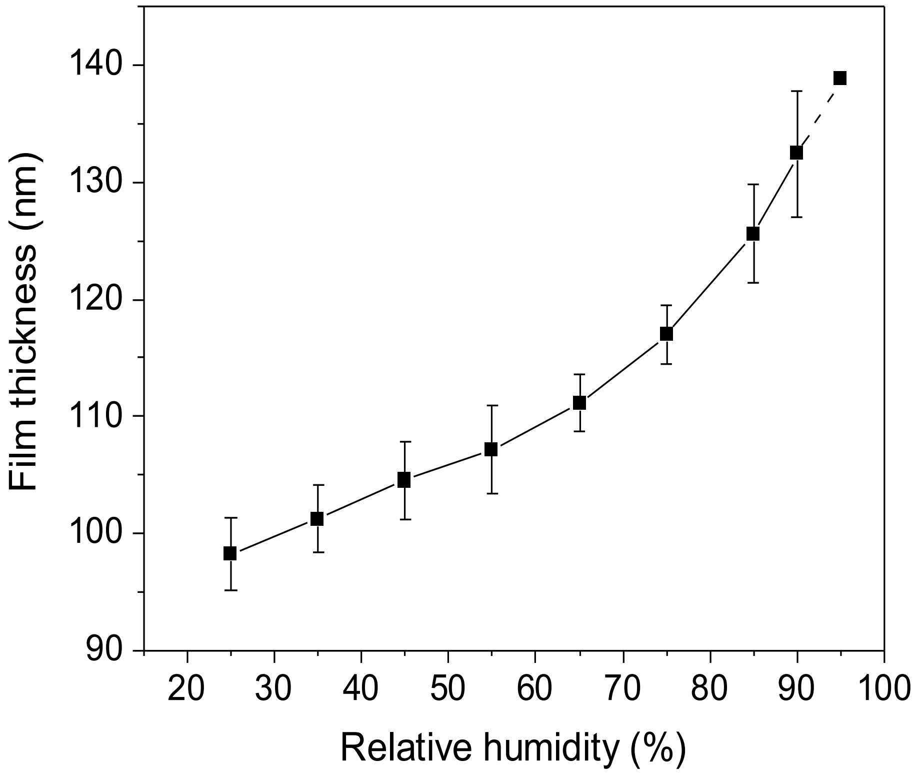

Supplementary Fig. 12 | Film thickness of PBBNB$^+$Br$^-$ thin film from 25% RH to 90% RH.

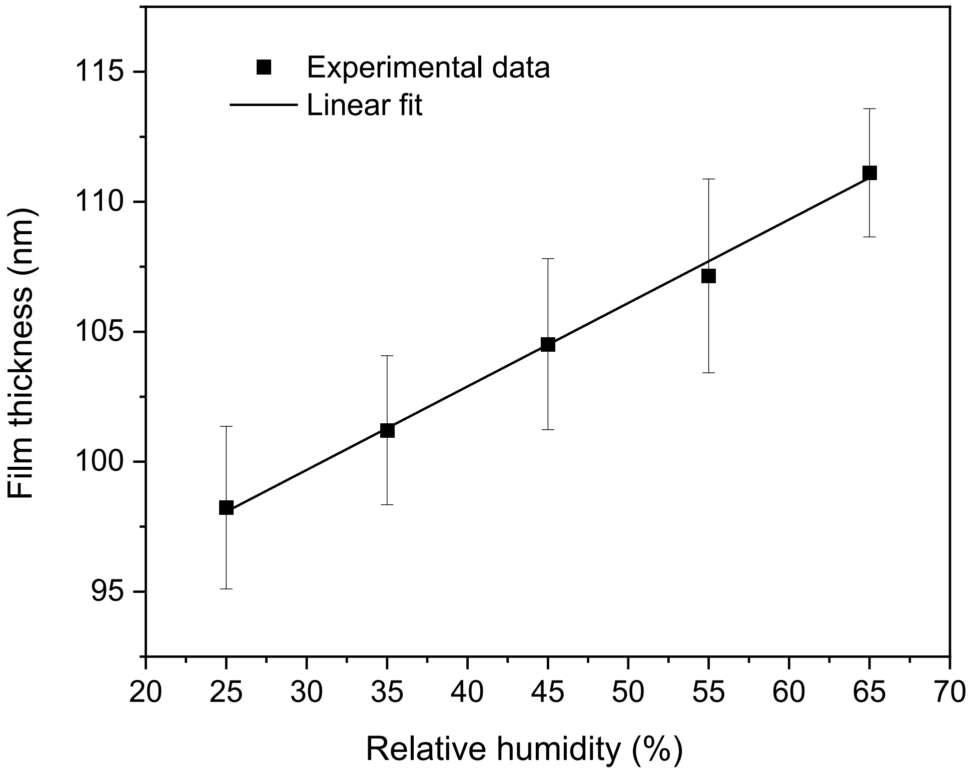

Supplementary Fig. 13 | Film thickness of $PBBNB^+Br^-$ thin film from 25% RH to 65 % RH. The film thicknesses of $PBBNB^+Br^-$ thin films below 65% RH were fit to a linear function.

The linear function is as follows.

$\text{Film thickness} = 90.05 + 0.321 \times \text{RH}$, $R^2 = 0.99753$

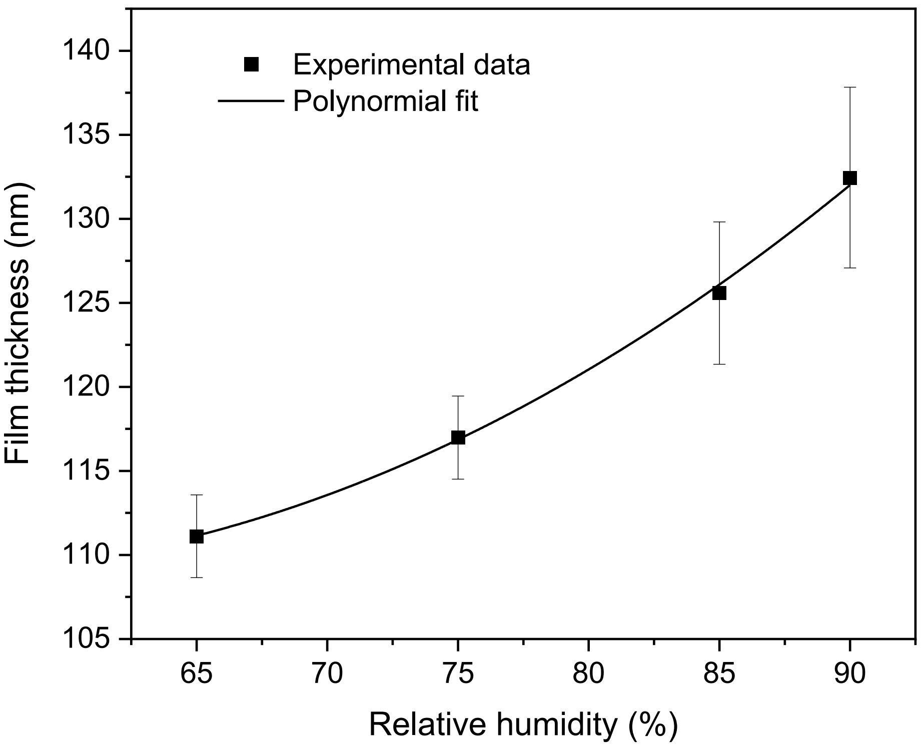

Supplementary Fig. 14 | Film thickness of PBBNB$^+$Br$^-$ thin film from 65% RH to 90 % RH. Film thicknesses of PBBNB$^+$Br$^-$ thin films above 65% RH were fit to a polynomial function. The film thickness at 95% RH was extrapolated to be 138.8 nm using the polynomial function.

The polynomial function is as follows.

$\text{Film thickness} = 0.0175 \times \text{RH}^2 - 1.88 \times \text{RH} + 159.36$, $R^2 = 0.99869$

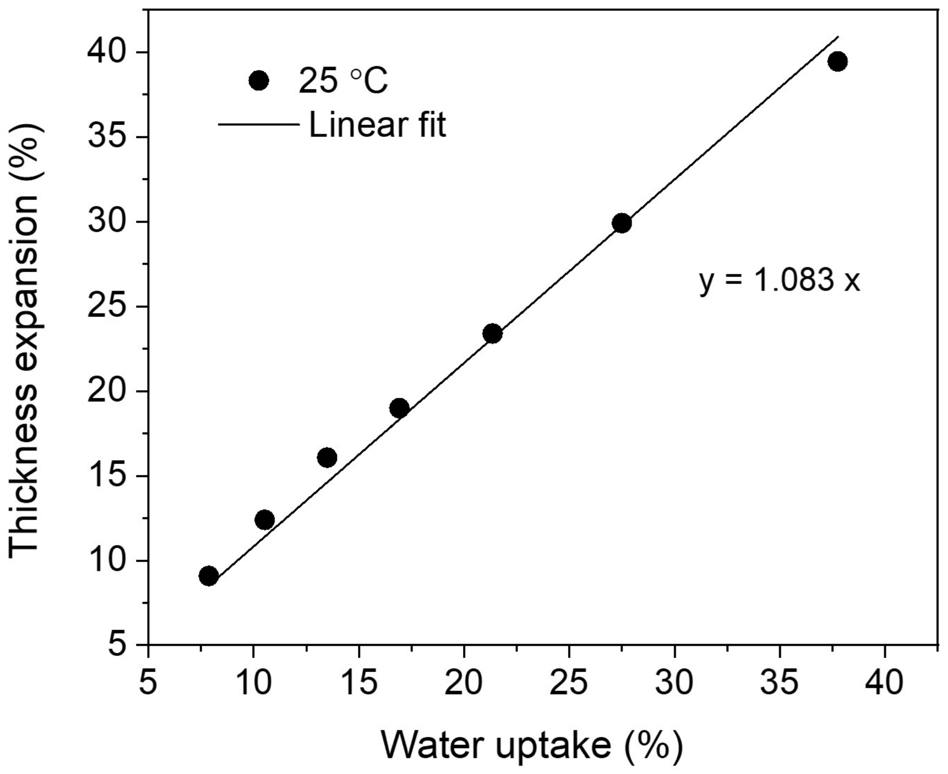

Supplementary Fig. 15 | Thickness expansion of PBBNB$^+$Br$^-$ as a function of water uptake. Water uptake (WU) = $\frac{W_w}{W_P}$; Thickness expansion = $\frac{\Delta h}{h_P}$, where W_w is the weight of water molecules in the film, W_P is the weight of the dry PBBNB$^+$Br$^-$ film, Δh is the film expansion due to the addition of water molecules, and h_P is the thickness of the dry PBBNB$^+$Br$^-$ film. The linear relationship between the thickness expansion and water uptake indicates that the volume expansion of the PBBNB$^+$Br$^-$ is due to the added volume of water molecules in the film and our calculations for water volume fraction (ϕ_w) from thickness measurements shown below are valid.

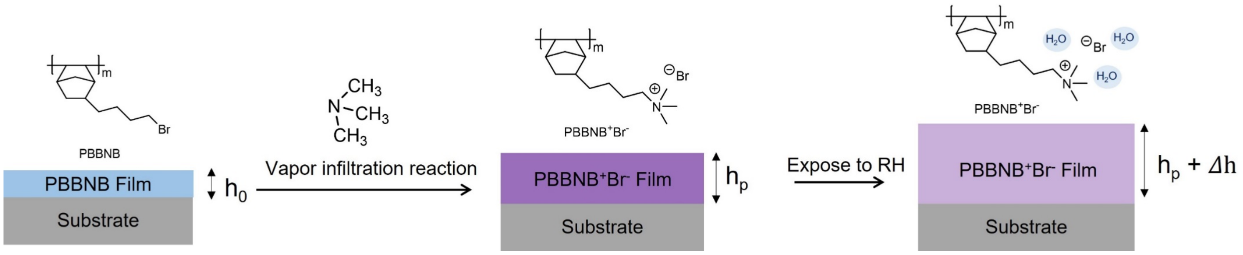

Supplementary Fig. 16│Schematic illustration of calculating water volume fractions in $PBBNB^+Br^-$ films. PBBNB film has a film thickness of h_0 after being spin coated onto the substrate. When we convert neutral PBBNB film to charged $PBBNB^+Br^-$ film, the thickness expands to h_p. If we expose the film to a humidified environment, the film thickness will further increase to $h_p + \Delta h$. If we assume the volume increase of the film is due to the added volume of water molecules. Then, the water volume fraction (ϕ_w) can be calculated by equation (5).

Volume of dry polymer (V_p) = substrate area (S) × h_p

Volume of polymer and water ($V_P + V_w$) = S × ($h_p + \Delta h$)

Water volume fraction (ϕ_w) $= \frac{V_w}{V_w+V_P} = \frac{S \times \Delta h}{S \times \Delta h + S \times h_P} = \frac{\Delta h}{\Delta h + h_P}$ (5)

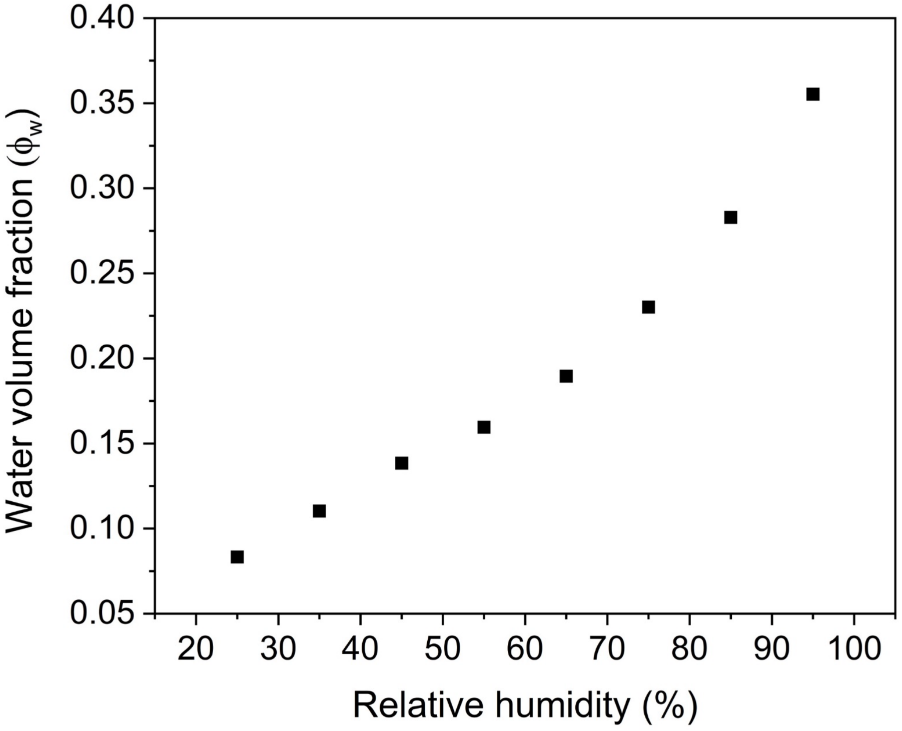

Supplementary Fig. 17 | Water volume fraction of the AEM as a function of RH.

S2.4 Thermal behaviors of polynorbornene polymers

Segmental mobility of polymer backbone contributes to the site hopping mechanism for ion transport and it is necessary to measure the glass transition temperatures (T_g) of $PBBNB^+Br^-$ polymers at different hydration levels. Thermalgravimetric analyzer (TGA) was used to determine the degradation temperature for $PBBNB^+Br^-$ polymers (see Supplementary Fig. 18&19), and differential scanning calorimetry (DSC) measurements were used to measure the T_g of the polymer (see Supplementary Fig. 20).

Before reacting with TMA, the onsite degradation temperature of neutral PBBNB is 362 °C (Supplementary Fig. 18). After the quaternization reaction, the charged $PBBNB^+Br^-$ first lost 11 wt% of mass due to the evaporation of absorbed water from room temperature to *ca.* 120 °C and after 286 °C, $PBBNB^+Br^-$ went through two steps of degradation (51 wt% weight loss and 36 wt% weight loss). Given to the results from TGA, DSC experiment with scanning temperature from 0 °C to 300 °C was performed on PBBNB and the T_g was not observed in this range (Supplementary Fig. 20a). For $PBBNB^+Br^-$, a needle was used to poke a small hole on an aluminum hermetic DSC pan and the sample was held at 180 °C to remove the absorbed water from ambient environment under nitrogen flow. T_g was not observed for $PBBNB^+Br^-$ from 0 °C to 260 °C for dry sample. (Supplementary Fig. 20 b&c) Since water works as a plasticizer, T_g of $PBBNB^+Br^-$ might shift to a detectable temperature at the most hydrated state[15]. To verify that, $PBBNB^+Br^-$ was placed in a hermetic DSC pan and the pan was held in a humidified chamber at 95% RH for two hours. The DSC pan was then closed with a hermetic lid inside of the chamber to prevent the dehydration of the polymer. DSC was performed for this hydrated sample, and there was no T_g observed from room temperature to 95 °C (Supplementary Fig. 20d), indicating that

even at 95% RH, the hydrated $PBBNB^+Br^-$ sample remained glassy. This also suggests that all EIS measurements were performed on glassy $PBBNB^+Br^-$.

To probe whether there is any free water in $PBBNB^+Br^-$, we performed DSC for $PBBNB^+Br^-$ at different hydration states. (see Supplementary Fig. 21) At 95% RH, the melting peak position of water is observed at – 8 °C, indicating that all water molecules are polarized and there is no bulk like water[16]. The peak position shifts to – 15 °C at 85% RH, and the peak area decreases compared to that at 95% RH, indicating that the water molecules are more polarized at 85% RH. The absence of the melting peak at 75% RH demonstrates that water molecules at this state are strongly polarized. The DSC measurements agree well with the calculations from radial distribution function (*g(r)*) (discussed in simulation section) and the *in situ* IR absorption spectrum, as shown in Fig 2.

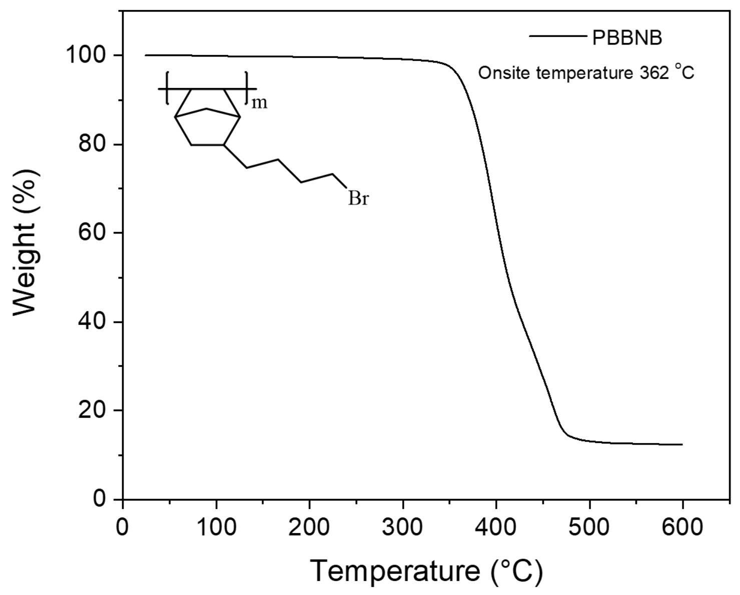

Supplementary Fig. 18 | Thermalgravimetric analyzer measurement of neutral poly (bromo butylnorbornene) (PBBNB).

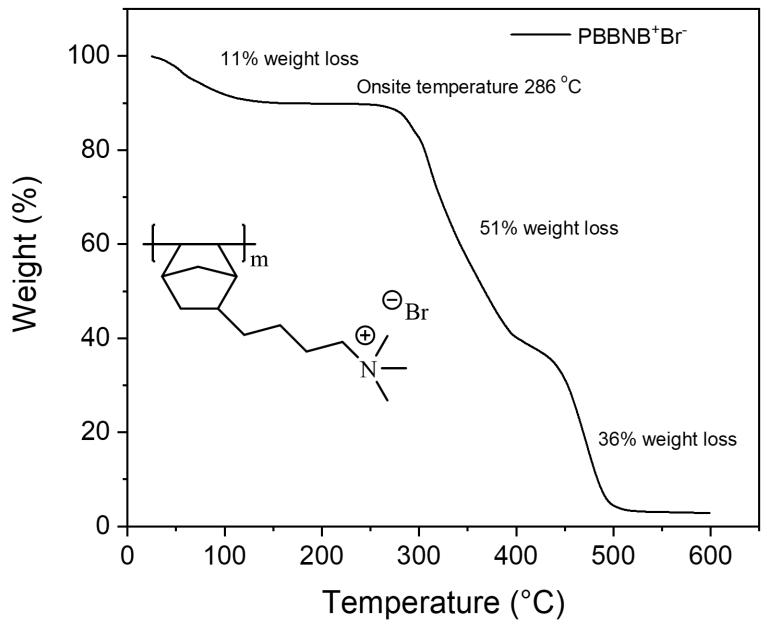

Supplementary Fig. 19 | Thermalgravimetric analyzer measurement of poly (bromo butylnorbornene) after functionalization ($PBBNB^{+}Br^{-}$).

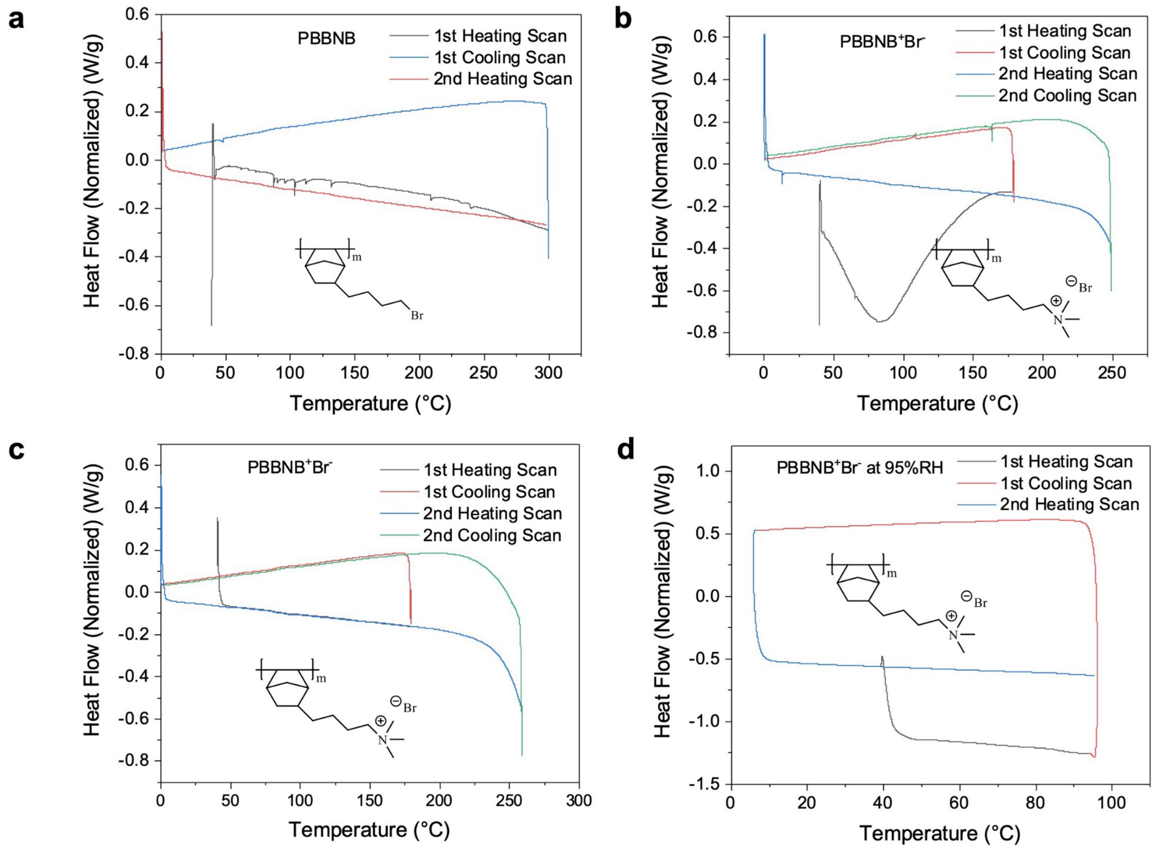

Supplementary Fig. 20│ Differential scanning calorimetry (DSC) measurements of polynorbornene based materials. The chemical structures are inserted in DSC Figureures. The heating and cooling scan rate is 10 °C/min. **a,** DSC traces of neutral poly (bromo butylnorbornene) (PBBNB) scanning from 0 °C to 300 °C, **b**, DSC traces of $PBBNB^+Br^-$ scanning from 0 °C to 250 °C. **c,** DSC traces of $PBBNB^+Br^-$ ranging from 0 °C to 260 °C. **d,** $PBBNB^+Br^-$ was held in an aluminum hermetic DSC pan and was pretreated at 95% relative humidity chamber with hermetic lid open. The sample was equilibrated in the humidity chamber for two hours. Hermetic pan was then closed inside the humidity chamber to reserve absorbed water molecules by cationic functional group. DSC measurements were performed to the sample ranging from 0 °C to 95 °C.

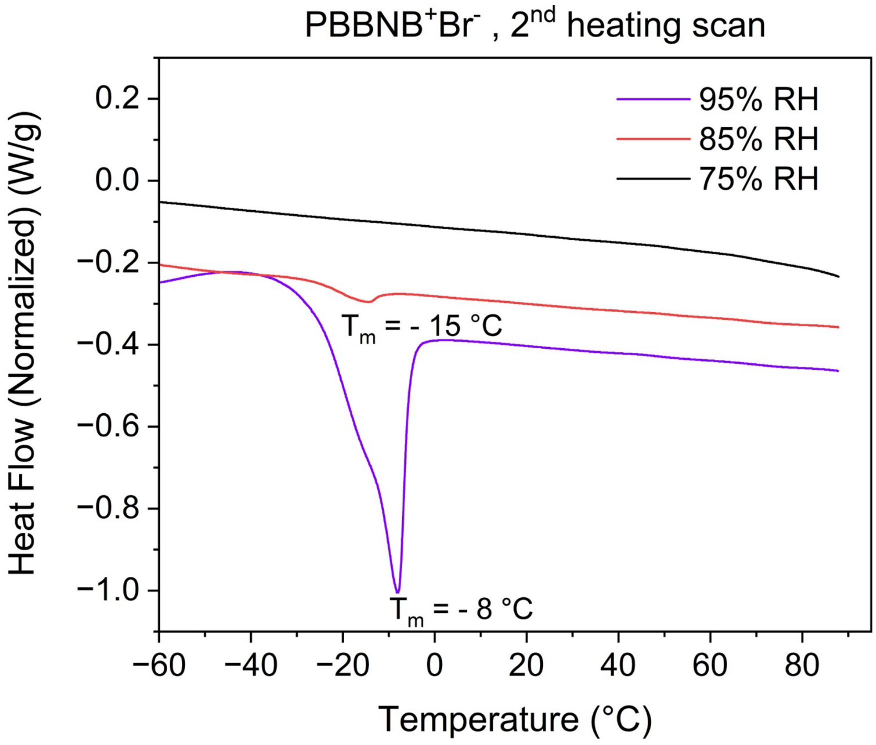

Supplementary Fig. 21 | DSC measurements of PBBNB$^+$Br$^-$ at 75% RH, 85% RH, and 95% RH.

S2.5 Ion conductivity measurements in $PBBNB^+Br^-$ anion exchange thin films

Supplementary Fig. 22 a shows an illustrative picture of the top and side views of the IDEs. During the measurement, a small sinusoidal (AC) voltage of known amplitude and frequency was imposed to our polymer film. The ratio and phase relation of the AC voltage and current signal response is the complex impedance *Z(jw)*, where *w* is frequency and *j* is the imaginary operator. In our case, the result of an impedance spectroscopy experiment is a data set in which ionic conductivity can be extracted via application of a physically reasonable equivalent circuit. Exemplary Nyquist plots with inserted equivalent circuit are shown in Supplementary Fig. 22b. For $PBBNB^+Br^-$ anion exchange thin films at different RH values, the Nyquist plots consist of one semicircle and a diffusive tail, indicating only ion conduction in $PBBNB^+Br^-$ films. The diameter for the semicircle corresponds to the ionic resistance in the film (R_{ion}).

For example, the $PBBNB^+Br^-$ film measured at 25 °C at 45% RH has a larger diameter than the sample measured at the same temperature but at a higher RH such as 85%. The equivalent circuit used to fit the Nyquist plot includes a resistor (R_s) in series with a resistor (R_{ion}) and a capacitor (C_{sub}) in parallel. R_s refers to resistance from the experimental set up, R_{ion} describes the resistive response from the film, and C_{sub} describes the capacitance of the silicon dioxide substrate. Constant phase elements (*CPEs*) are added in the equivalent circuit model in place of a capacitor to compensate for non-homogeneity in the system. CPE_{film} corresponds to the non-uniform transport behavior of the polymer film, and CPE_{int} corresponds to the behavior of the double-layer capacitance near the blocking electrodes. The conductivity of $PBBNB^+Br^-$ as a function of RH at 25 °C is shown in Supplementary Fig. 22c.

The geometry of the IDE and equation (6) used to calculate the conductivity are shown below.

$$\sigma = \frac{1}{R_{ion}} \frac{1}{(N-1)l} \frac{d}{h} \quad (6)$$

Where R_{ion} is the ionic resistance, d = 8 μm is the spacing between adjacent electrode teeth, l = 500 μm is the length of the electrode, N = 80 is the number of electrodes, h is the thickness of the film. The thickness of the film as a function of RH is measured by the *in situ* ellipsometer.

To understand ion transport, percolation theory was applied to fit the conductivity data. The results are shown in Fig. 1d and Supplementary Fig. 23. Critical percolation threshold ϕ_c can be extracted from Fig 1d. and universal constant γ_D can be extracted from Supplementary Fig. 23. The percolation theory is shown in equation (7).

$$\sigma = \sigma_0(\phi_w - \phi_c)^{\gamma_D} \quad (7)$$

Where σ is the ionic conductivity measured from EIS, σ_0 is the inherent conductivity, ϕ_w is the water volume fraction, ϕ_c is the critical water volume fraction at the percolation threshold, and γ_D is a universal constant that depends on the spatial dimensions only.

Above ϕ_c, excess water volume fraction is defined as $(\phi_w - \phi_c)$. Supplementary Fig. 23 shows a log-log plot of σ versus $(\phi_w - \phi_c)$. The plot obeys linear correlation and is fitted to extract σ_0 and γ_D . σ_0 and γ_D are determined to be 47.32 mS/cm and 1.51, respectively. Kirkpatrick demonstrated that for a three-dimensional system with random orientation of minority phase, γ_D was reported to range from 1.3 to 1.7 with 1.5 as the most probable value[35]. Our experimental value of γ_D is almost the same as the theoretical value, suggesting that conducting phases in our $PBBNB^{+}Br^{-}$ system are perfectly randomly orientated.

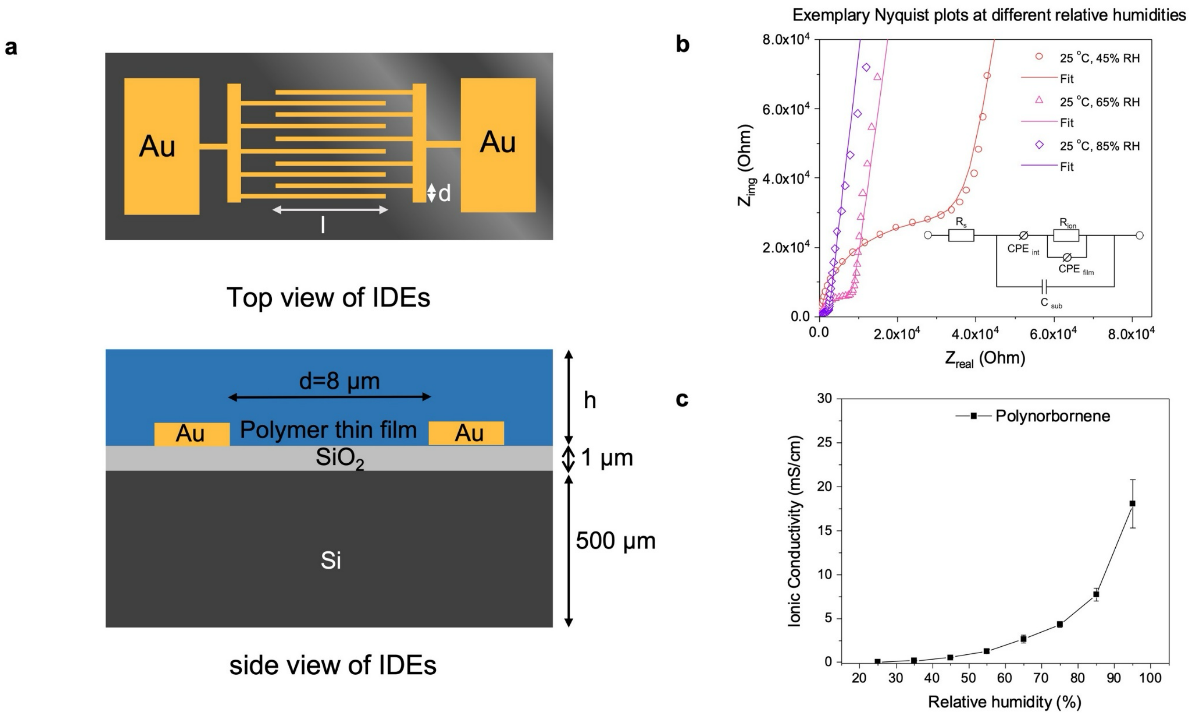

Supplementary Fig. 22│Ion conductivity measurements of the AEM films using interdigitated electrode arrays (IDEs) and EIS. a, Schematics of the top and side views of the IDE. **b**, Exemplary Nyquist plots of $PBBNB^{+}Br^{-}$ at 45% RH, 65% RH, and 85%RH. **c**, Ionic conductivity of Br^{-} in polynorbornene as a function of a RH at 25 °C.

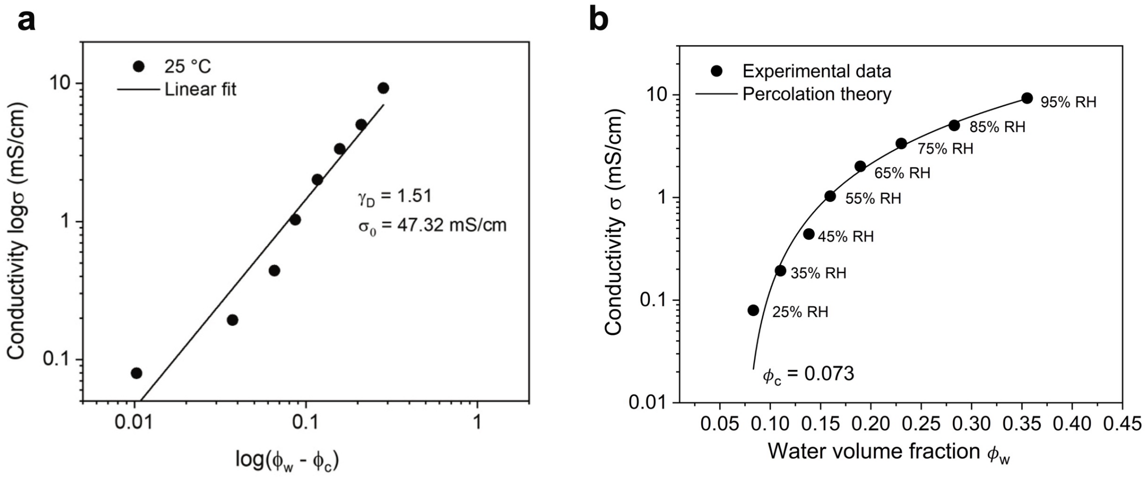

Supplementary Fig. 23│ Relationship between ionic conductivity and volume fraction. a, Ionic conductivity as a function of excess volume fraction (ϕ_w - ϕ_c). From the log-log plot, universal constant (γ_D) and inherent conductivity (σ_0) can be extracted from the linear fit. **b,** Ionic conductivities as a function of water volume fraction (ϕ_w). Percolation theory is used to calculate

the critical water volume fraction (ϕ_c). that describes the insulator-to-conductor transition of the AEM.

Supplementary Fig. 23a shows a log-log plot of σ versus $(\phi_w - \phi_c)$. The plot obeys linear correlation and is fitted to extract σ_0 and γ_D. σ_0 and γ_D are determined to be 47.32 mS/cm and 1.51, respectively. Below ϕ_c, water clusters are small and isolated and long-range ion transport is impossible. In our system, ionic conductivity of $PBBNB^+Br^-$ film is 0.08 mS/cm with ϕ_w of 0.083. Ionic conductivity will be even lower or hard to measure below ϕ_c.

Near or above the ϕ_c, the ionic conductivity (σ) follows a power law equation (8):

$$\sigma = \sigma_0 (\phi_w - \phi_c)^{\gamma_D} \quad (8)$$

Where σ_0 is the inherent conductivity, ϕ_w is the water volume fraction, ϕ_c is the critical water volume fraction at the percolation threshold, and γ_D is a universal constant that depends on the spatial dimension only.

Supplementary Fig. 23b shows the semi-log plot of σ as a function of ϕ_w. The semi-log plot follows a power law equation (8) and ϕ_c is determined to be 0.073. ϕ_c works as an indicator for insulator-conductor transition in an ion conducting polymer. However, percolation theory does not describe the transition of ion transport mechanisms and can not provide molecular-level insights about the onset of Br^- transport in regime II (>55 %RH).

S2.6 X-Ray scattering of PBBNB$^+$Br$^-$ anion exchange materials

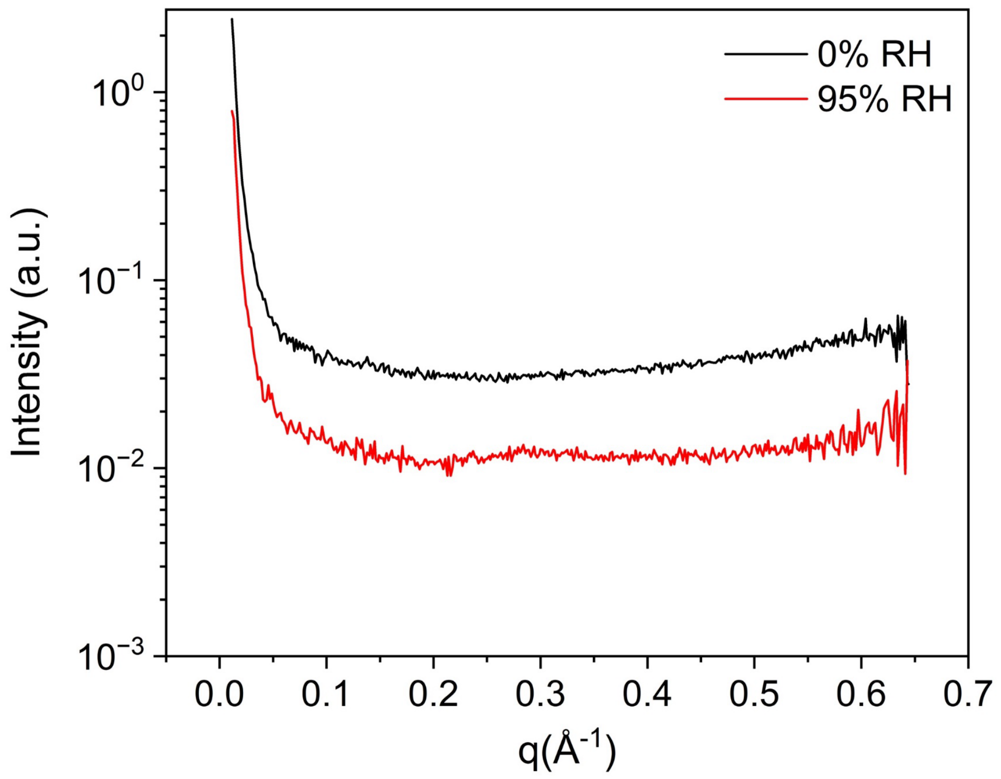

Supplementary Fig. 24 | SAXS data of PBBNB$^+$Br$^-$ at 0% RH and 95% RH.

Supplementary Section 3: Simulation Methods

All simulated systems consist of 25 chains with 40 monomers for each chain. The degree of functionalization which refers to the fraction of quaternary ammonium groups is 100%. Water content is taken from experiments corresponding to the relative humidity of (0%, 25%, 35%, 45%, 55%, 65%, 75%, 85%, 95%). To begin with, nine independent copies of a simulation box with dimensions of 15 × 15 × 15 nm^3 were created. All 25 polymer chains are generated randomly in the simulation cell and 1000 Br^- ions were added to the cell to neutralize the positively charged,

cationic groups in the system. Then, water molecules were added to each system to achieve the corresponding water content.

With the initial configurations set up, for each system, the energy was first minimized via steepest descent to remove any steric clashes. The resulting configurations were relaxed and equilibrated at 500 K and 1 atm for 30 ns with a 1 fs time step. A Nosé-Hoover thermostat was used to couple temperature and a Berendsen barostat was used to couple pressure. Then the systems were cooled from 500 K to 298 K with a cooling rate of 10 K/1 ns followed by 30 ns of equilibration run. Subsequently, the simulation was performed with Parrinello-Rahman pressure coupling for 30 ns; an average density of the system was computed by using the last 15 ns of simulation. Finally, an NVT production run of 150 ns was performed for each system with computed density.

S3.1 Force Field Parameters and Simulation Details

In this section, we provide the parameters used to perform the molecular dynamics (MD) simulations. The force field parameters for the bromide anion and quaternary ammonium groups were taken from the work by Padua[17]. As a modification of the nonpolarizable force field, the partial charges of ionic groups and ions were scaled by a factor of 0.8 to account for the effect of electronic dielectric screening of Coulombic interactions[18]. The all-atom optimized potentials for liquid simulations (OPLS-AA) force field were used for all inter- and intramolecular interactions. The total energies for the system were evaluated as a sum of individual energies for bond stretching, angle bending, dihedral torsion terms for the bonded interactions, and Coulomb, and 12-6 Lennard-Jones terms for the nonbonded interactions. The SPC/E water model was used in all hydrated systems.

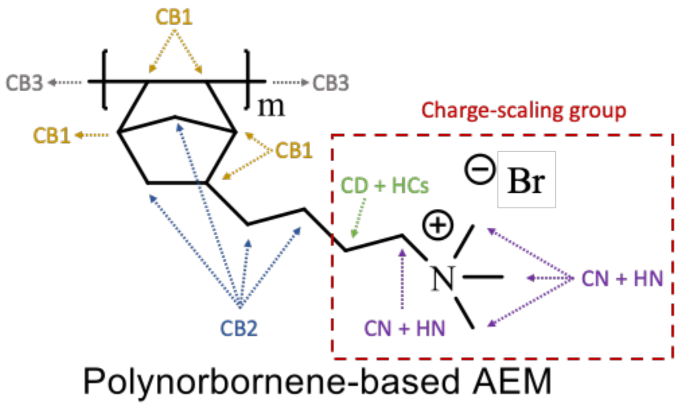

Supplementary Fig. 25 | Reference labels for atom types in force field parameters. All hydrogen atoms without specified is HC.

Non-bonded Interaction Parameters

Non-bonded interactions were computed intermolecularly and for intramolecular atom pairs separated by four or more bonds in the form of

$$E_{nonbond} = \sum_{i}\sum_{j>i}\left\{4\epsilon_{ij}\left[\left(\frac{\sigma_{ij}}{r_{ij}}\right)^{12} - \left(\frac{\sigma_{ij}}{r_{ij}}\right)^{6}\right] + \frac{q_i q_j}{4\pi\epsilon_0 r_{ij}}\right\}$$

where σ_{ij} and ϵ_{ij} are Lennard-Jones radii and well-depths, q is the partial charge and r_{ij} is the separation distance. Geometric combining rules are utilized:

$$\sigma_{ij} = \sqrt{\sigma_{ii}\sigma_{jj}} \text{ and } \epsilon_{ij} = \sqrt{\epsilon_{ii}\epsilon_{jj}}$$

Charges and 12-6 Lennard-Jones parameters used in the simulation are given in Table S2 and include the 0.8-scaled charges.

Supplementary Table 2: Non-bonded potential parameters

atom	m(amu)	σ_{ii}(Å)	ϵ_{ii}(kcal/mol)	q(e)
CB1	12.011	3.50	0.066	-0.060
CB2	12.011	3.50	0.066	-0.120
CB3	12.011	3.50	0.066	-0.180
CD	12.011	3.50	0.066	0.008
CN	12.011	3.50	0.066	-0.136
HC	1.008	2.50	0.030	0.060
HCs	1.008	2.50	0.030	0.048
HN	1.008	2.50	0.030	0.104
N	14.007	3.25	0.170	0.096
Br^-	79.904	3.97	0.206	-0.800

Bonding Potential Parameters

Energy for the bond stretching is the sum of harmonic potential between two covalently bonded atoms

$$\mathrm{E_{bond}} = \sum_i k_{r,i}\left(r_i - r_{0,i}\right)^2$$

where k_r is the bonding force constant and r_0 represents the bond distance at equilibrium. The parameters used for this interaction are given in Table S3.

Supplementary Table 3: Bonding potential parameters

bond	$k_r\left(\frac{\text{kcal}}{\text{mol}\cdot\text{Å}^2}\right)$	r_0(Å)
C-C**	268	1.529
C-N*	382	1.448
H-C**	340	1.090

C represents any type of carbon atom, and H* represents any type of hydrogen atom.*

Bending Potential Parameters

Angle bending terms consist of bond-angle vibration between a triplet of atoms represented via a harmonic potential

$$E_{\text{angle}} = \sum_i k_{\theta,i}\left(\theta_i - \theta_{0,i}\right)^2$$

where k_θ is the bending force constant and θ_0 represents the angle at equilibrium. The parameters used for this interaction are given in Table S4.

Supplementary Table 4: Bending potential parameters

bend	$k_\theta\left(\frac{\text{kcal}}{\text{mol}\cdot\text{rad}^2}\right)$	θ_0(degrees)
C-C*-C**	58.35	112.70
C-C*-H**	37.50	110.70
H-C*-H**	33.00	107.80

H-C*-N*	35.00	109.50
C-N4-C**	51.80	107.20
C-C*-N*	56.20	109.47

C represents any type of carbon atom, and H* represents any type of hydrogen atom.*

Torsion Potential Parameters

Torsional energetics are represented by a series of cosine potential

$$E_{torsion} = \sum_i \frac{1}{2}\left[V_{1,i}(1 + cos\,\phi) + V_{2,i}(1 - cos\,2\phi) + V_{3,i}(1 + cos\,3\phi) + V_{4,i}(1 - cos\,4\phi)\right]$$

where V are Fourier coefficients and ϕ is the dihedral angle. The parameters used for this interaction are given in Table S5.

Supplementary Table 5: Torsional potential parameters

torsion	$V_1\left(\frac{\text{kcal}}{\text{mol}}\right)$	$V_2\left(\frac{\text{kcal}}{\text{mol}}\right)$	$V_3\left(\frac{\text{kcal}}{\text{mol}}\right)$	$V_4\left(\frac{\text{kcal}}{\text{mol}}\right)$
C-C*-C*-C**	1.300	-0.050	0.200	0.000
H-C*-C*-H**	0.000	0.000	0.300	0.000
C-C*-C*-H**	0.000	0.000	0.300	0.000
H-C*-C*-N*	-1.013	-0.709	0.473	0.000
H-C*-N-C**	0.000	0.000	0.560	0.000
C-N4-C*-C**	0.416	-0.128	0.695	0.000
C-C*-C*-N*	2.392	-0.674	0.550	0.000

C represents any type of carbon atom, and H* represents any type of hydrogen atom.*

System Parameters for Initial and Equilibrated Configurations

This section provides detailed information on the hydration number (λ) and the equilibrium density of the simulated systems. The hydration number is calculated based on the water content measured in the experiments (see Section S2.3 for detailed experimental information). To demonstrate that the system is at equilibrium, we monitored the density as a function of simulation time. The system was considered to be at equilibrium when the density exhibited no drift and the average density remained unchanged over a sufficiently long simulation period (see Section S3.1).

Supplementary Table 6: Hydration Numbers and Densities of the Systems

RH(%)	Hydration Number (λ)	Equilibrium Density (g/cm^3)
25	1.41	1.161 ± 0.004
35	1.88	1.167 ± 0.004
45	2.41	1.168 ± 0.003
55	3.03	1.172 ± 0.003
65	3.82	1.169 ± 0.003
75	4.92	1.167 ± 0.003
85	6.76	1.160 ± 0.003
95	13.06	1.130 ± 0.002

The density initially increases and then decreases with increasing water molecules, which is consistent with previous studies[1, 20]. In membranes with low hydration, voids are present within the structure. Initially, at lower hydration levels, these voids are occupied by water, leading to a

rise in system density before any significant change in overall volume occurs. After these voids are mostly filled, additional water absorption causes the membrane to expand, which results in a reduction in density at higher λ values.

S3.2 MSD and Diffusivities

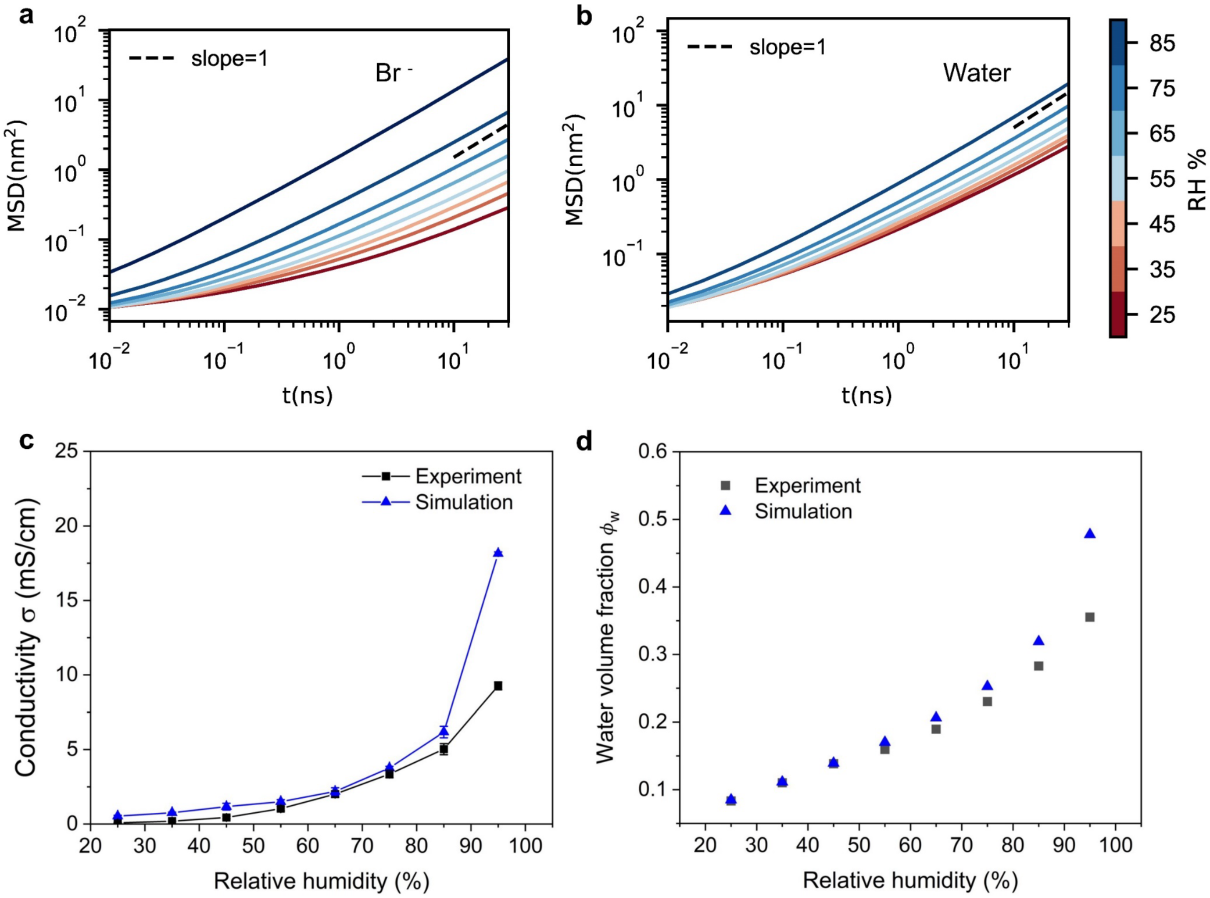

Supplementary Fig. 26 | Comparisons between simulation and experimental data. a-b, Mean square displacement (MSD) at 298 K in MD simulations for various RH levels of **(a)** Br^- anions and **(b)** water. **c-d,** Comparison of simulation and experimental data for **(c)** conductivity and **(d)** water volume fraction.

Supplementary Fig. 26 presents comparative analysis between our MD simulations and experimental results. There is an excellent alignment between these two data with respect to both conductivity and water volume fractions. In order to calculate conductivity in the simulation, we

begin by measuring the mean square displacement (MSD) of Br^- in each system in Supplementary Fig. 26a. MSD of water is also presented for reference in Supplementary Fig. 26b. Supplementary Fig. 26a demonstrates that above 65% RH, the MSD long-time slopes on a log-log scale approach unity and exhibit sufficient linearity to be classified within the diffusive regime. Consequently, we can calculate the self-diffusion coefficient D from the MSD, using Einstein's relation

$$D = \frac{1}{6} \lim_{t\to\infty} \frac{d}{dt} < \left(r(t) - r(t_0)\right)^2 >$$

From 25% to 65% RH systems, the log-log MSD slopes are less than unity. Thus, diffusion coefficients were approximated by apparent diffusivities which are evaluated from 15 ns to 135 ns averaged over all time origins in the trajectory.

Supplementary Table 7: Br^- diffusion coefficients and MSD slopes

RH(%)	$D(10^{-5}\mathrm{cm}^2/\mathrm{s})$	log-log MSD slope
25	0.0038 ± 0.0004	0.74
35	0.0055 ± 0.0003	0.78
45	0.0087 ± 0.0017	0.84
55	0.0115 ± 0.0010	0.85
65	0.0174 ± 0.0020	0.87
75	0.0315 ± 0.0010	0.95
85	0.0562 ± 0.0035	0.93
95	0.2132 ± 0.0013	0.97

Diffusion coefficients and slopes are fitted from 15 ns to 135 ns.

S3.3 Ionic Conductivity

Ionic conductivity σ was calculated to compare with experimental results in Supplementary Fig. 26c. From the Green-Kubo equation, we can relate microscopic charge current in simulation with ionic conductivity

$$\sigma = \frac{1}{6k_BTV} \lim_{t\to\infty} \frac{d}{dt} < \sum_i \sum_j q_i\, q_j [r_i(t) - r_i(0)] \cdot [r_j(t) - r_j(0)] >$$

where q is the charge of species and V is the volume of the simulation box.

The conductivity consists of five types of contribution for the system contains ions[19]: cation-self (σ_{cat}^s), anion-self (σ_{an}^s), cation-distinct (σ_{cat}^d), anion-distinct (σ_{an}^d), and cation-anion-distinct ($\sigma_{cat,an}^d$) conductivities.

$$\sigma = \sigma_{cat}^s + \sigma_{an}^s + \sigma_{cat}^d + \sigma_{an}^d + 2\sigma_{cat,an}^d$$

Note that cation in our system is N^+ in the quaternary ammonium group which is tethered to the polymer. Since the polymer is glassy, and the local bond relaxation is very slow, the σ_{cat}^s term can be neglected. From calculation. the three distinct conductivity terms are three orders of magnitude less than σ_{an}^s, thus can also be neglected. Finally, ionic conductivity can be approximated by

$$\sigma \cong \sigma_{an}^s = \frac{1}{6k_BTV} \lim_{t\to\infty} \frac{d}{dt} < \sum_{i_{an}} q_{i_{an}}^2 \left(r_{i_{an}}(t) - r_{i_{an}}(0)\right)^2 >$$

$$\sigma \cong \frac{NDq^2}{k_BTV}$$

where N is the total number of Br^- in the box and D is the diffusion coefficients in Table S7.

Supplementary Fig. 26d shows comparison of water volume fraction as a function of RH from experiments and simulation. Overall, the water volume fraction and ionic conductivities of the AEM obtained from experiments and simulation show great agreement, indicating valid force field parameters used in MD.

S3.4 Polymer Dynamics

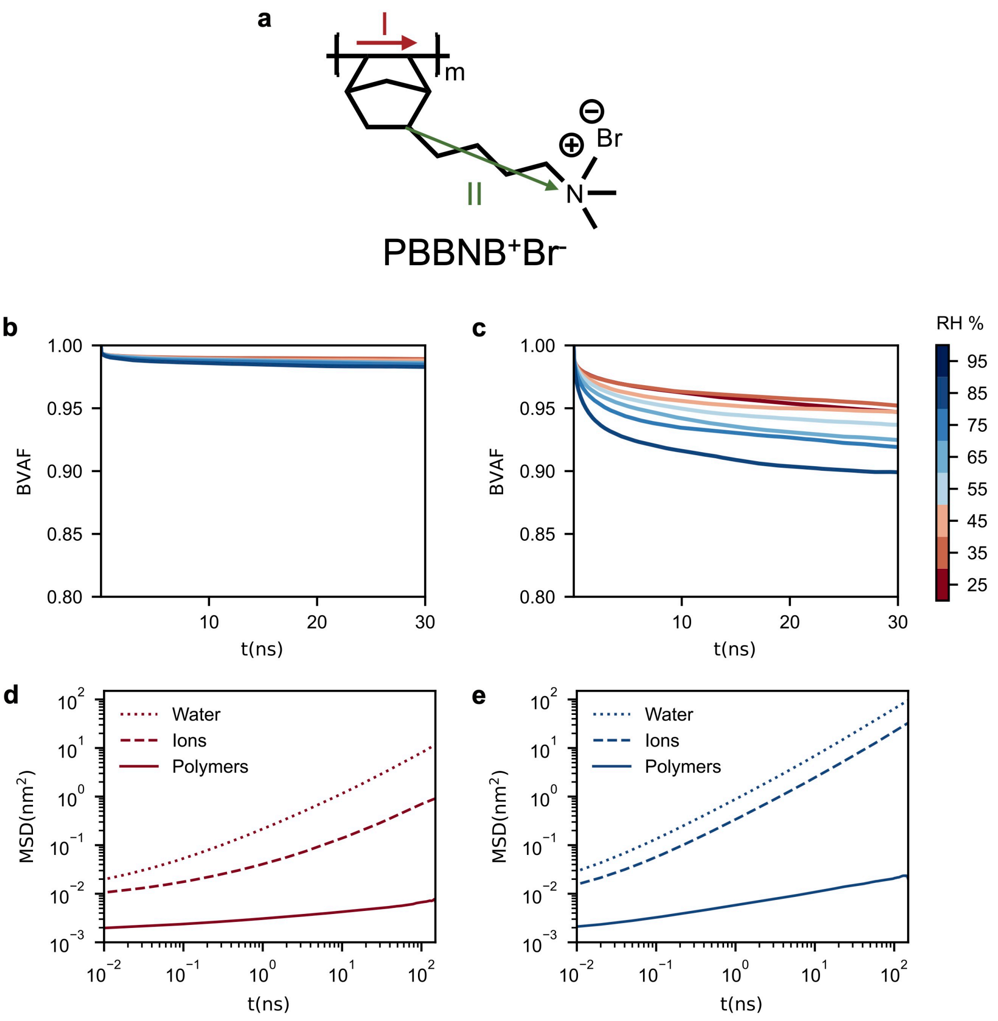

Supplementary Fig. 27 | Polymer dynamics characterized using the bond vector autocorrelation function (BVAF) and mean square displacement (MSD). a, Schematic illustrating two types of bond vectors used in calculations. **b-c,** BVAF for the **(b)** backbone bond and **(c)** sidechain-terminal vector. **d-e,** MSD of polymer atoms, bromide ions, and water molecules at **(d)** 25% RH and **(e)** 85% RH.

All experimental systems were maintained at room temperature, which is below the glass transition temperature of the polymer. To quantify the segmental mobility of the polymers, we computed the bond vector autocorrelation functions (BVAF) at varying length scales. The BVAF is defined as follows:

$$\mathrm{BVAF}(t) = \langle b(t) \cdot b(0) \rangle$$

where b is the vector connecting two specified atoms. In our study, we have defined two types of vectors, which capture relaxation phenomena at the polymer backbone level and at the sidechain reorientation level. Supplementary Figs. 27b-c demonstrate that both types of vectors, across all systems, exhibit slow relaxation dynamics. This observation confirms that all our systems exhibit slow polymer dynamics at room temperature.

To compare the polymer dynamics to those of the water and ions, we calculated the MSD of polymer atoms, bromide ions, and water molecules across all RH levels. Supplementary Figs. 27d-e demonstrate that, at both low and high hydration levels, the MSD of polymers is three to four orders of magnitude lower than that of the ions and water. This indicates that, across the entire range of RH investigated in our work, polymer dynamics are sufficiently slow to be considered negligible compared to the dynamics of ions and water.

S3.5 Radial Distribution Functions

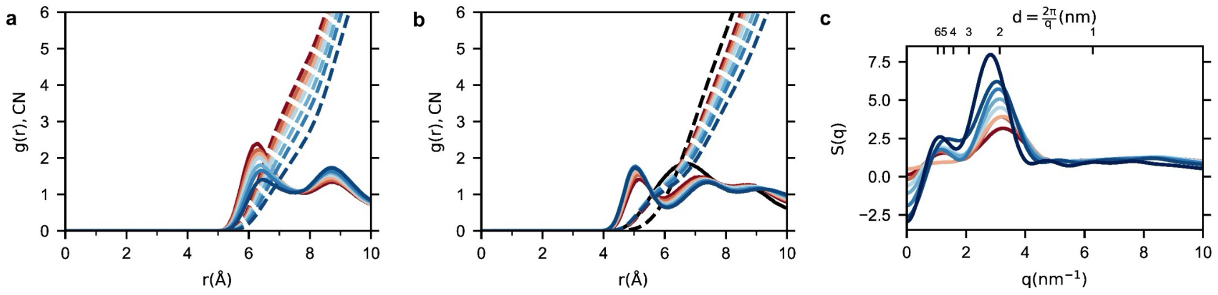

Supplementary Fig. 28│Radial distribution functions g(r) (solid lines) and coordination numbers CN (dashed lines) at different RH for **a,** N^+ - N^+, **b,** Br^- - Br^-. **c,** Structure factors of water. The legend for color codes can be found in Fig 2 in the main text and Supplementary Fig. 25.

Supplementary Fig. 28a-b showcase the radial distribution functions (RDFs) for both N^+-N^+ and Br^--Br^- at various RH levels. As RH increases, the interactions between ammonium groups weaken, suggesting that the distance between polymer chains expands and the resulting channels widen. To investigate the spatial distribution of water molecules and their behavior within various hydrophilic domains, we computed water-water structure factors from the Fourier transform of the Ow-Ow RDFs, as illustrated in Supplementary Fig. 28c. The structure factor S(q) was calculated with the following equation:

$$S(q) = 1 + 4\pi\rho \int_0^\infty \frac{\sin(qr)}{qr} r^2 [g(r) - 1]\, \mathrm{d}r$$

In this expression, q represents the magnitude of the wave vector, and rho indicates the number density of water in the simulation box. These structure factors reveal a shift in the second peak from larger q in low RH systems to smaller q in high RH systems, indicating larger cluster formation as RH increases. The narrower peaks in high RH systems further substantiate this observation.

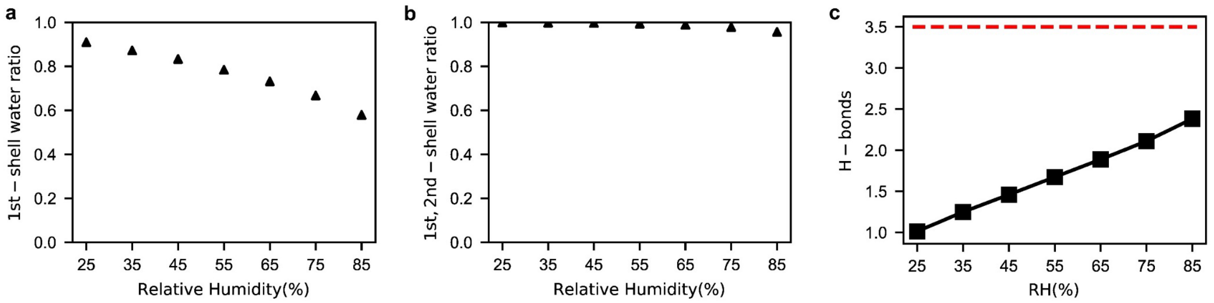

Supplementary Fig. 29 | Analysis of water distribution and hydrogen bonding across RH levels. a, Ratio of water in the first shell to total water in the system. **b**, Combined ratio of water in the first and second shells to total water. **c**, Number of H-bonds per water molecule at varying RH levels, with dashed red line as reference for bulk water H-bonds.

Our examination extends to water populations within the first two solvation shells, as shown in Supplementary Fig. 29. Supplementary Fig. 29a reveals the proportion of water in the first shell relative to the total water in the system. Supplementary Fig. 29b provides the ratio of water in the first and second shells to the total water in the system. These data confirm that the vast majority of water resides in the first two shells—at unity for low RH systems and above 95% at the highest RH levels—affirming the absence of bulk-like water across all systems.

Supplementary Fig. 29c provides a detailed account of the number of hydrogen bonds (H-bonds) per water molecule across all RH levels, with bulk water H-bonds as a reference. In every RH system, the average number of H-bonds per water molecule falls below that in bulk water, reflecting a disrupted global H-bond network and a lack of bulk-like water even at the highest RH levels.

S3.6 Percolation analysis by MD

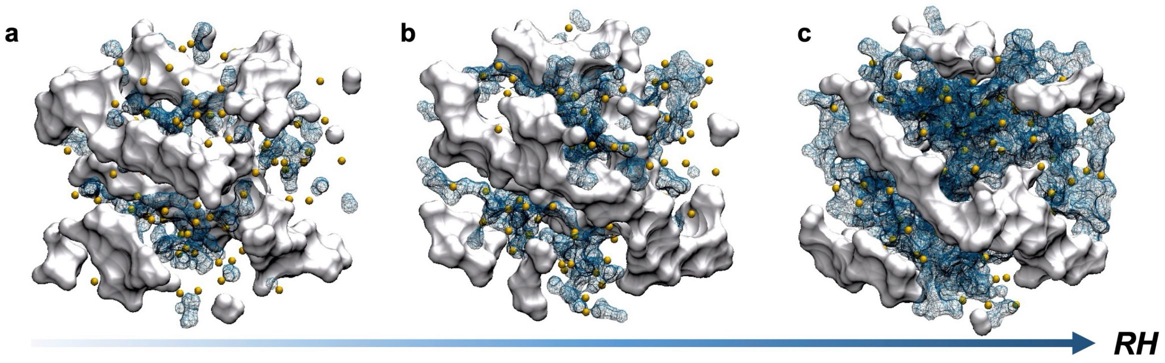

Supplementary Fig. 30 | MD simulation snapshots illustrating the system at various RH levels. a-c, 3D overviews at **(a)** 25%, **(b)** 55%, and **(c)** 85% RH, providing a comprehensive view of percolation. Grey surfaces represent polymers, brown spheres are Br-, and the blue areas indicate water.

Supplementary Fig. 30 showcases 3D and 2D snapshots of the system clusters. The percolation probability and cluster count definitions can be found in the main text. Specifically, Supplementary Fig. 30a-c provide additional snapshots at higher RH levels, supplementing Figure 3 in the main text.

Supplementary Fig. 31a clarifies the calculation of percolation probability: the system's largest cluster is marked in red, while smaller or medium-sized clusters are depicted in blue. The percolation probability is computed as the ratio of the red cluster's area to the total area of both red and blue clusters.

Sparse clusters can be observed at a low RH level (RH=25%) in Supplementary Fig. 30a. As Supplementary Fig. 31b confirms, systems at this RH level contain many small-sized clusters, indicating that the ion and water molecule clusters are isolated. In contrast, at higher RH levels (starting from 55% RH), most clusters interconnect as shown in Supplementary Fig. 30b. Even though the system contains few clusters, it includes a remarkably large one, approaching 100% in

size (Supplementary Fig. 31b). Particularly, at 85% RH, a complex percolation network emerges (Supplementary Fig. 30b), and a single cluster includes all anions and water molecules (Supplementary Fig. 31a-b). This suggests complete interconnection of anions and water molecules throughout the system.

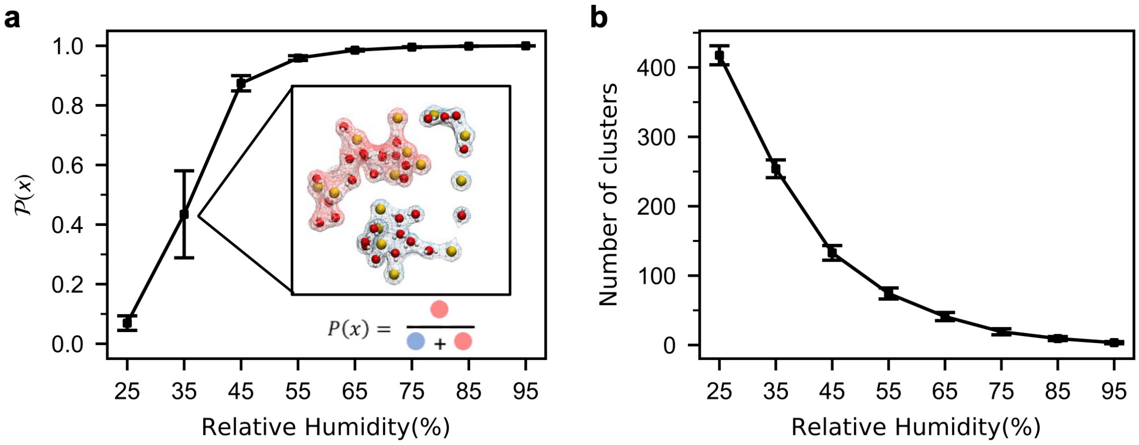

Supplementary Fig. 31 | Cluster and percolation analysis across varying RH systems. a, Calculation of percolation probability based on identified clusters, with an inset outlining the calculation method. **b,** Count of clusters present in each system.

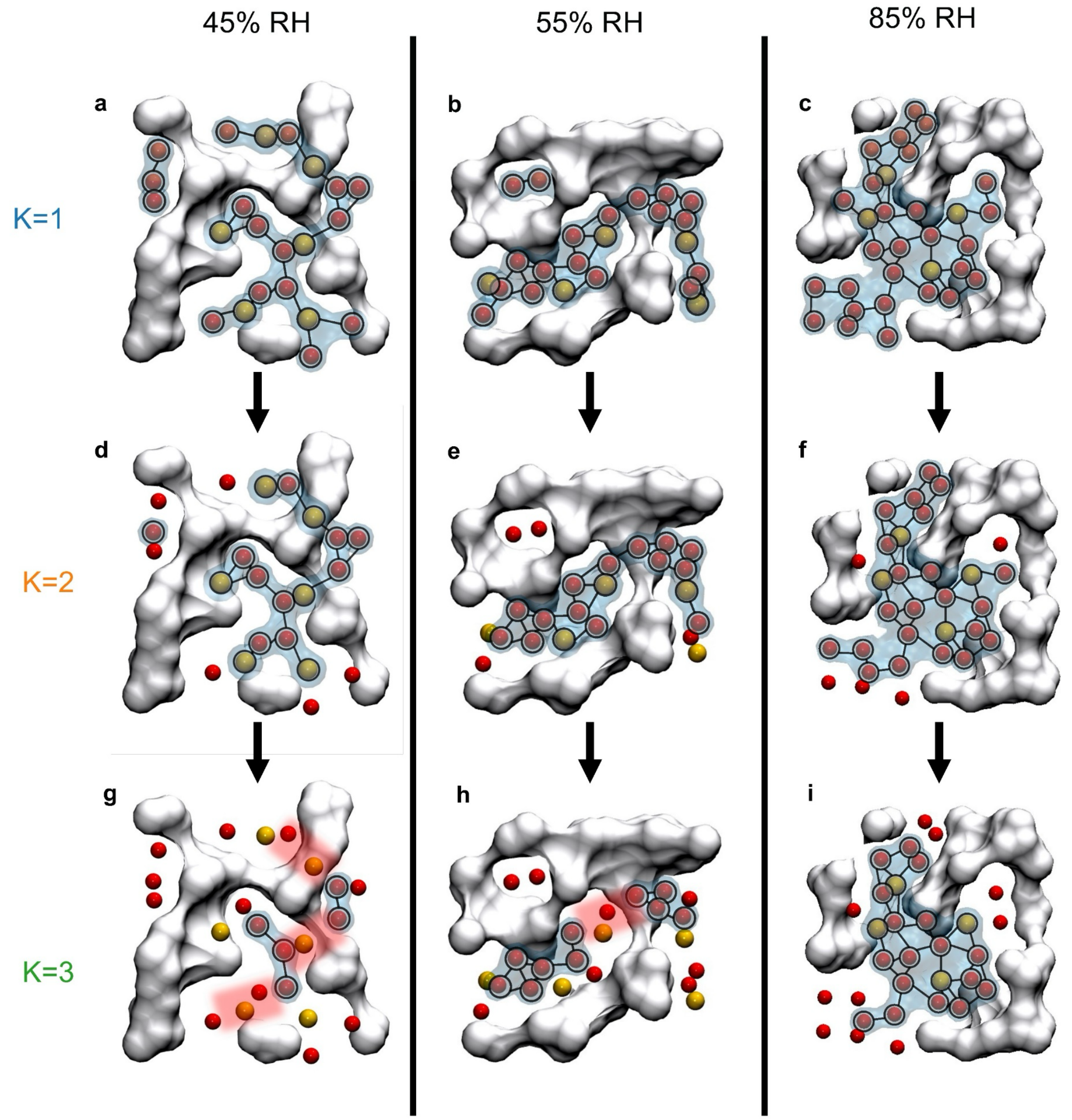

Supplementary Fig. 32 │ Robustness of percolation in the ion-water network analyzed by graph theory. *k-stub* analysis snapshots are shown at **(a-c)** $k = 1$, **(d-f)** $k = 2$, and **(g-i)** $k = 3$ for the polymer system at 45%, 55%, and 85% RH, respectively. Nodes (black circles, O_w or Br) and edges (black lines, H-bonds) illustrate the ion-water network.

Supplementary Fig. 32 details a stepwise k -stub analysis, and includes a comparison with a 55% RH case. At $k = 1$, the system is in its original state, and the calculated percolation probability aligns with the results from Supplementary Fig. 31a. This is demonstrated in systems at 45%, 55%, and 85% RH, where clusters, along with their associated nodes and edges, are illustrated.

When k is elevated to 2, nodes with only one edge are excluded from the clusters. These nodes are often found at the corners of the clusters. As anticipated, the percolation probability remains largely unchanged, because these molecules' removal does not significantly influence the percolation network. This trend is consistent across all the systems displayed at 45%, 55%, and 85% RH.

Increasing k to 3 leads to further node removal under more stringent criteria: nodes with one or two edges are removed, leaving only those with a minimum of three edges. Despite this, a robust water percolation network continues to persist in the 85% RH system, unaffected by bottlenecks that may impede ion mobility.

In contrast, for the 45% RH system, the removal of these critical nodes, marked in red in the accompanying visualizations, results in the fragmentation of the largest cluster into smaller ones. This significant reduction in nodes corresponds with a marked decrease in percolation probability, approaching levels seen in low RH systems. The 55% RH system showcases intermediate behavior between the 45% and 85% RH systems, with the removal of key nodes and bottlenecks (indicated in red) leading to noticeable changes in the percolation network and corresponding probability.

Supplementary Section 4: 2D IR

S4.1 Experimental and simulated 2D IR analysis

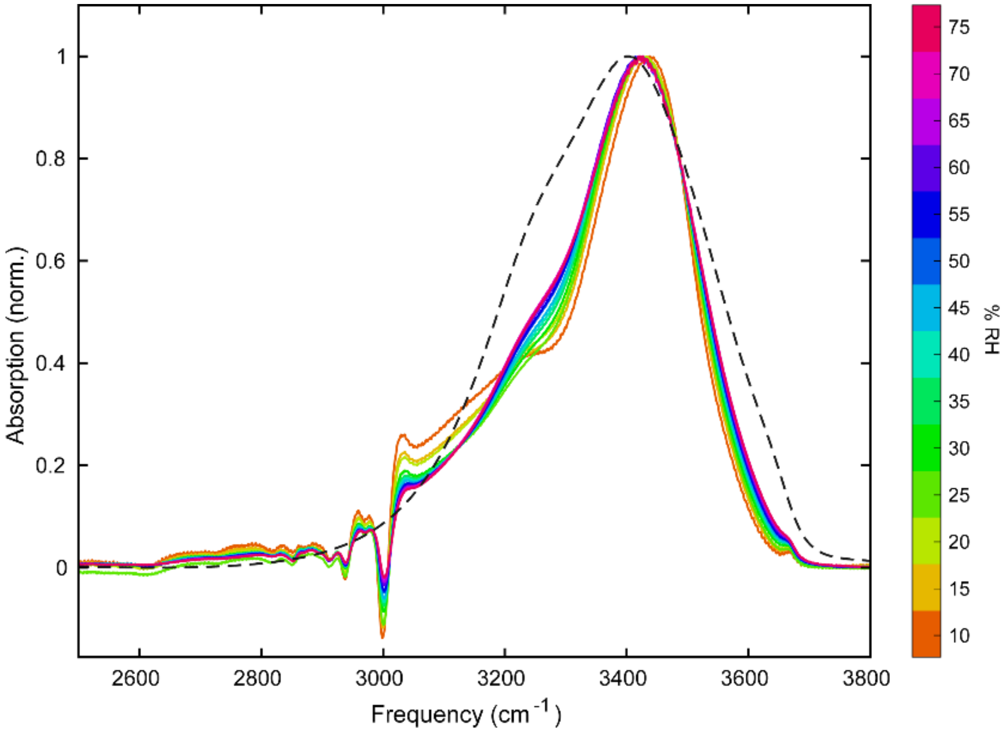

Supplementary Fig. 33 | FTIR spectra of H_2O OH stretch as a function of RH. Absorption spectra of the OH stretching mode of H_2O in the bulk liquid (blacked dashed line) and as a function of RH in the polymer. The 0% RH spectrum is used as the reference to subtract the absorption from the polymer.

In Supplementary Fig. 33 we show the *in situ* IR absorption spectra of the OH stretching vibration in H_2O molecules, without isotopic dilution. As with the OD stretch of HOD the water molecules in the polymer show a significantly narrowed and blueshifted absorption as compared to the bulk liquid, at all RH. This corroborates the interpretation of the OD stretch, that there is no bulk-like water hydrogen bonding network in the polymer. As the RH is increased the peak absorption slightly redshifts and broadens, which is consistent with the formation of hydrogen bonds, but nevertheless indicative of a highly disrupted network that is far from the bulk liquid.

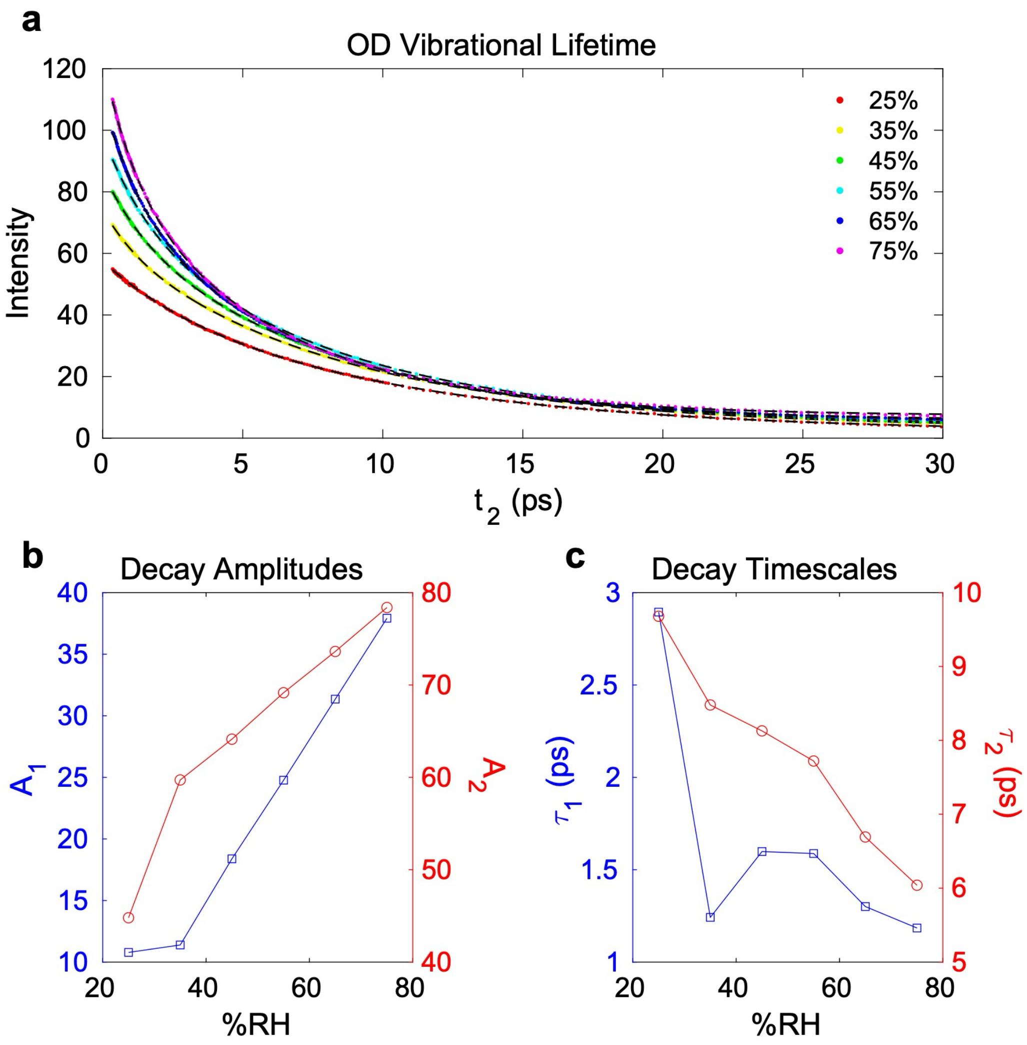

Supplementary Fig. 34 | OD stretch vibrational lifetime. a, TA decay traces for the peak of the signal of the OD stretch mode as a function of RH showing the changes in the signal intensity and vibrational lifetime. The dashed lines show biexponential fits to the data. **b,** Amplitudes and **c,** timescales for the two decay components as a function of RH.

In Supplementary Fig. 34 we show the vibrational lifetime kinetics measured from the isotropic TA as a function of RH. We see that as the water content of the polymer increases so does the initial magnitude of the signal. Simultaneously, the lifetime of the vibrational excitation decreases with the RH. This is shown by the parameters determined from biexponential fits to the data, which show both time constants accelerating with RH, and the relative weight of the slow component

compared to the fast component decreasing. Even at 75% RH the relaxation is significantly slower than the bulk liquid, but the trend is approaching the bulk behavior.

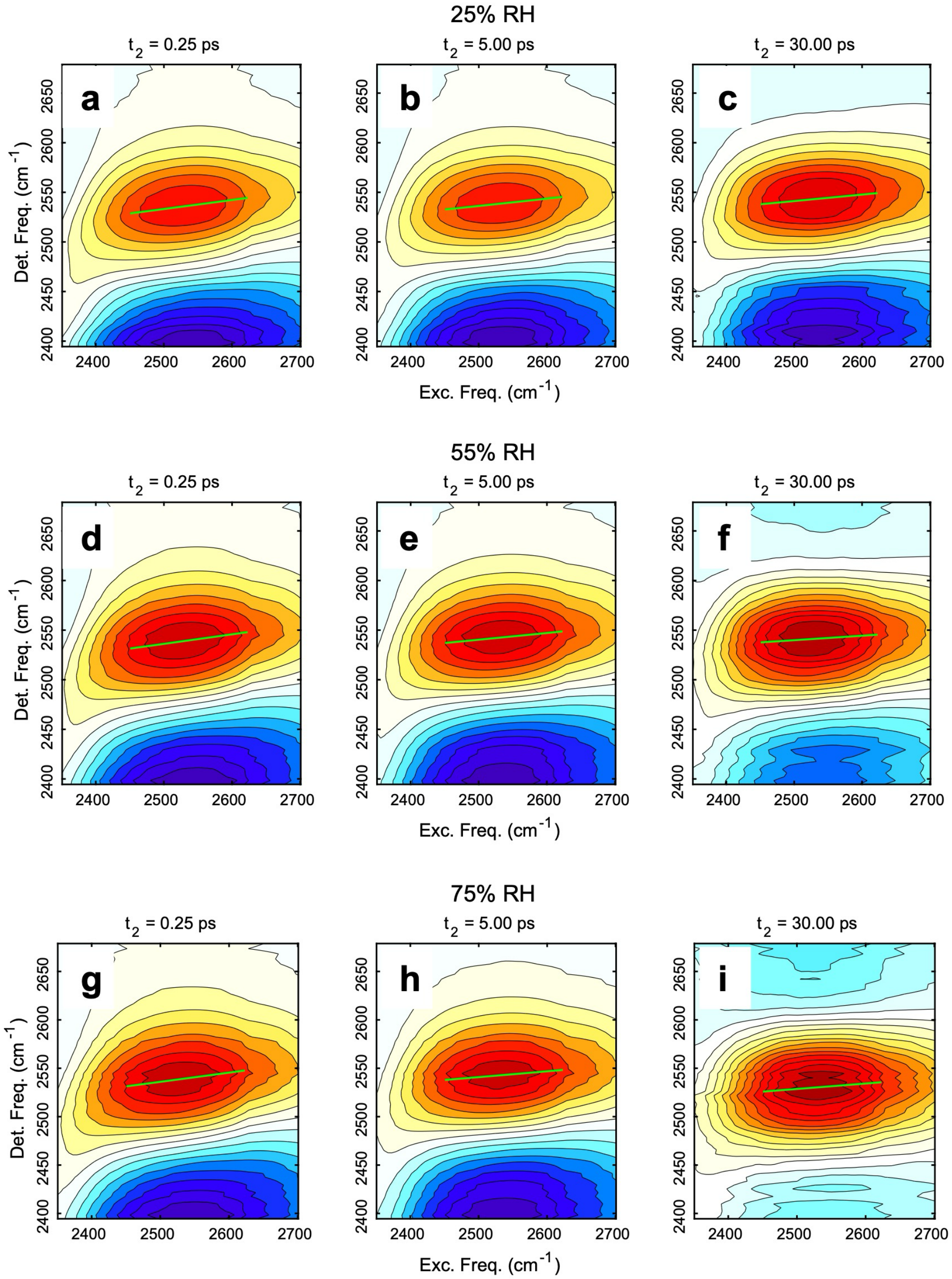

Supplementary Fig. 35 | 2D IR spectra of OD stretch as a function of RH and t_2. Select 2D IR spectra at waiting times t_2 = 0.25, 5 and 30 ps. **a-c,** 25% RH, **d-f,** 55% RH, **g-i,** 75% RH. The 0-1 transitions are shown in the yellow-red contours and the 1-2 transitions are shown in the blue contours. The green lines indicate the center lines used to extract the CLS decays.

In Supplementary Fig. 35 we some select 2D IR spectra of the OD stretching mode in HOD as a function of waiting time and RH. These spectra show how the slope of the center line, shown in green, decays with increasing t_2, with the kinetics of this decay accelerating with RH. At long waiting times the spectrum begins to take on the appearance of the hot ground state for the high RH condition, while for the low RH condition it maintains the appearance of a typical vibrational excitation. This indicates the partial formation of a hydrogen bonding network at high RH, as the intermolecular modes of the network give rise to the hot ground state spectrum. Even at high RH the low intensity of the hot ground state and the residual slope of the center line demonstrate the hydrogen bonding network is far from the bulk liquid.

S4.2 Analysis for ion transport mechanisms

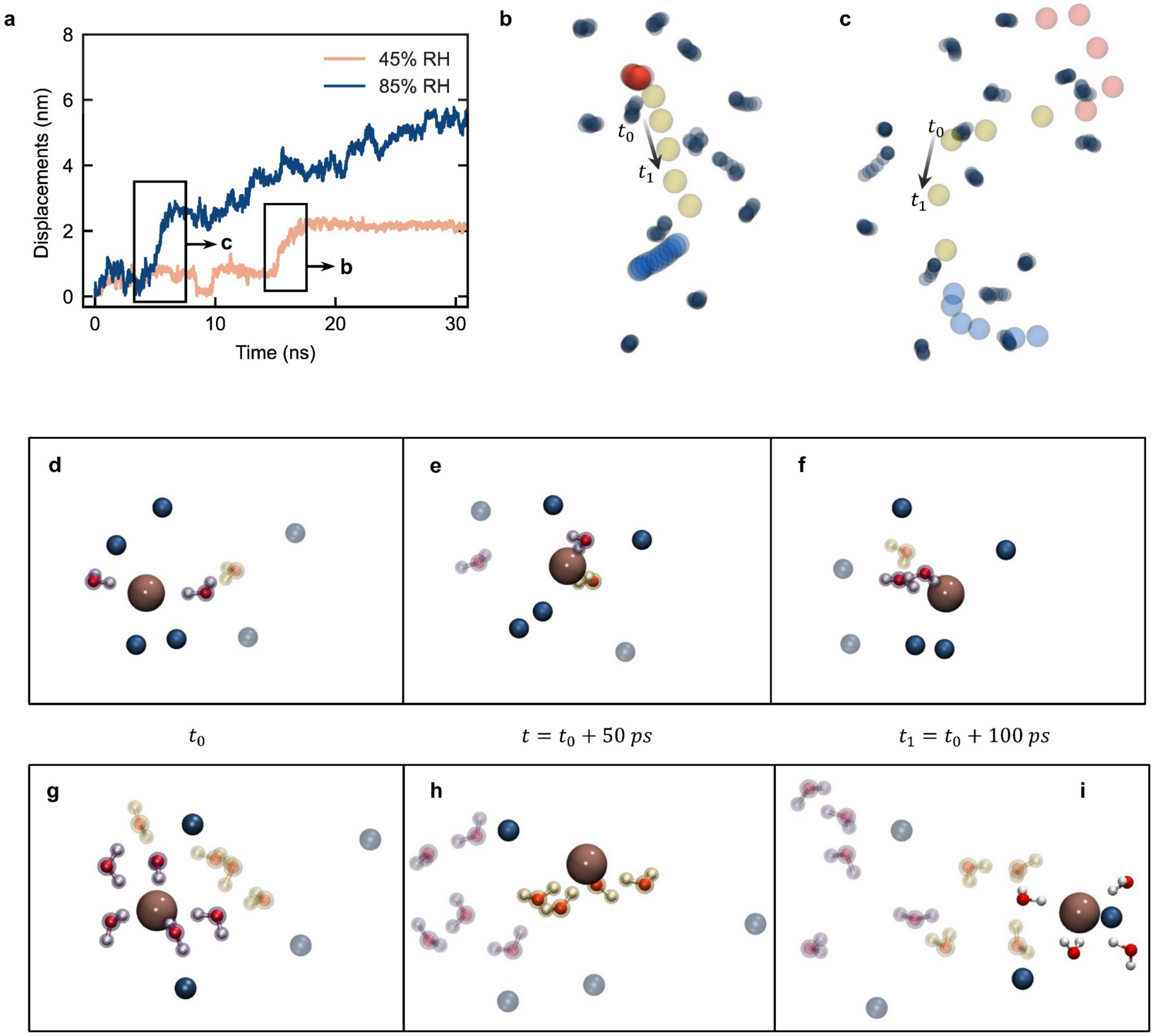

Supplementary Fig. 36 │ Two ion transport mechanisms. a, Displacement trajectories of representative ions over a 30 ns simulation period at 45% and 85% RH. Highlighted trajectories (in black boxes) are further expanded in panels (**b**) and (**c**). **b- c,** Overlapped 5 ns frames of selected ion trajectories from orange line and blue line in panel (**a**), respectively. Trajectory colors indicate three equal time intervals: red (beginning), yellow (middle), and blue (end). N^+ ions are shown in dark blue. **d-I,** Specific snapshots from the trajectories in (**b**) and (**c**) at three different time points ($t = t_0$, $t = t_0 + 50\, ps$, $t = t_1 = t_0 + 100$ ps) illustrate ion motion through N^+ solvation sites. Solid N^+ and water molecules currently in the 1st solvation shell are in dark blue and red/white respectively, while those outside the shell are shown transparently. **d-f,** Snapshots from the trajectory in (**b**) at 45% RH: **(d)** At $t = t_0$, water molecules in the 1st shell are outlined in purple. **(e)** At the intermediate state, a yellow-outlined water molecule moves from the 2nd to the 1st shell for ion solvation as one purple-outlined molecule departs. **(f)** At $t = t_1$, the remaining purple-outlined molecule returns to the 1st shell while the yellow-outlined one exits. **g-i,** Snapshots from the trajectory in (c) at 85% RH: **(g)** At $t = t_0$, water molecules in the 1st shell are outlined in purple. **(h)** At the intermediate state, yellow-outlined molecules enter the 1st shell, replacing exiting purple-outlined ones. **(i)** At $t = t_1$, yellow-outlined molecules leave the 1st shell, substituted by new water molecules (no outlines), as original purple-outlined ones diffuse further.

Supplementary Fig. 36 presents a compelling depiction of two distinct ion transport mechanisms. The figure begins with the trajectories of representative ions over a 30 ns simulation period at 45% and 85% RH, as displayed in Supplementary Fig. 36a. Represented trajectories are highlighted and further expanded in Supplementary Fig. 36b-c, with overlapped 5 ns frames to showcase the intricate ion movements. The trajectory colors mark three equal time intervals: red (beginning), yellow (middle), and blue (end).

The ensuing panels, Supplementary Fig. 36d-i, focus on the ion transport through N^+ solvation sites at different time points. Here, solid N^+ and water molecules currently in the 1st solvation shell are emphasized with color, while those outside the shell are shown transparently to capture the dynamics of solvation.

The trajectory at 45% RH (panels d-f) displays a mechanism of solvation exchange where one water molecule from the second shell moves into the first shell, replacing a departing molecule. At the final time point, the original molecule returns to the first shell, replacing the new one. This cycle of exchange and return demonstrates one mechanism of ion transport.

The trajectory at 85% RH (panels g-i), on the other hand, shows a different process. Here, new water molecules enter the first shell, replacing departing ones. In the final snapshot, new water molecules come in as the original ones diffuse further away.

It's clear from the presented data that the second ion transport mechanism relies heavily on the dynamics of water molecules in the 2nd solvation shell, as confirmed by IR, 2D IR and simulation data. Furthermore, this mechanism requires at least 3 edges or the overlap of the 2nd solvation shell, as evidenced by k -stub analysis. Together, these findings bring fresh insights into

the complexity and variability of ion transport mechanisms, which may be modulated by the conditions of the system.

S4.3 Decoupled reorientation dynamics of water molecules.

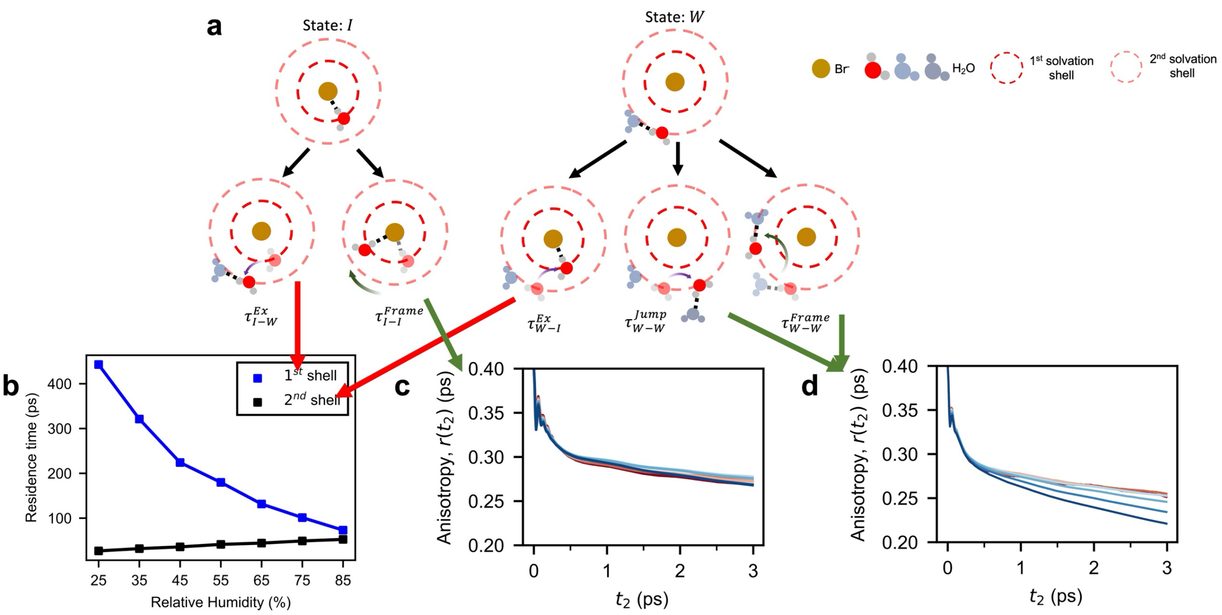

Supplementary Fig. 37│ Decoupled reorientation dynamics of water molecules. **a**, Schematic illustration shown in Fig. 4g, detailing reorientation motions in two initial states. **b**, Relaxation time of τ_{I-W}^{Ex} and τ_{W-I}^{Ex}. **c**, Relaxation time of τ_{I-I}^{Frame}. **d**, Relaxation time of τ_{W-W}^{Jump} and τ_{W-W}^{Frame}.

We monitored the water molecules according to their presence in either the first or second solvation shell. Upon transitioning between shells—from the first to the second or vice versa—we calculated their residence time, as displayed in Supplementary Fig. 37b. For water molecules remaining within the same solvation shell throughout the observation period, we assessed the reorientation dynamics by determining the anisotropy decay. These findings are presented in Supplementary Fig. 37c-d for water in the first-shell and second-shell residence, respectively. Notably, water molecules in the first solvation shell exhibit consistent behavior across all RH levels, evidenced by the overlapping curves. Concurrently, only water molecules in the second shell exhibit a trend similar to that shown in Fig. 4e-f, suggesting that the 2nd-shell water

significantly influences the two distinct regimes in orientational dynamics. Therefore, the mobility of ions is critically dependent on the water in the second solvation shell.

Supplementary Section 5: Additional validation

To further validate our findings, particularly in the low RH region, we conducted three additional independent MD simulations, starting each from scratch. For each RH level ranging from 25% to 45%, three samples were prepared using distinct initial configurations. Polymer chains and ions were randomly placed in a large simulation box with unique random seeds to ensure both uniqueness and reproducibility. Water molecules were then added to each system to achieve the corresponding water content, following the equilibrium and production protocols as described in the above sections.

Snapshots of the simulation box without water are shown in Supplementary Fig. 38, illustrating that each independent simulation begins with a distinct initial configuration. After the random addition of water, the densities of each system at the same RH level were found to be within the RMSD. The averaged values, along with error bars, are provided in Supplementary Table 6. Note: error bars are standard error obtained with n = 3 samples.

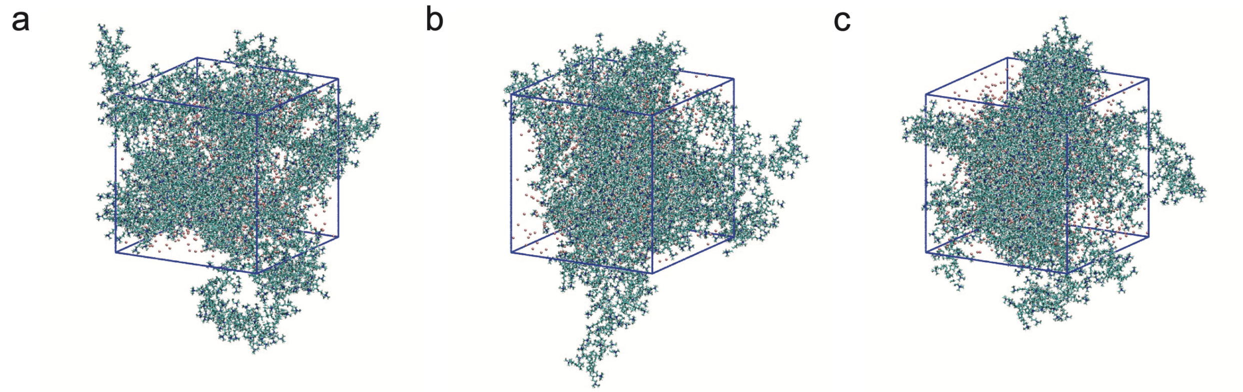

Supplementary Fig. 38 | Snapshots of three additional independent simulations starting from different initial polymer configurations.

To assess the robustness of our findings, several key analyses were performed for each RH level and each independent simulation. These analyses confirmed that the conclusions remained consistent across different initial configurations. Given that polymers in the low RH region are relatively immobile and could have a larger effect on the dispersion of water molecules, the consistency observed in the low RH region suggests that similar stability would be observed at higher RH levels. We attribute this consistency to the randomly distributed hydrophilic functional groups and the hydrophobic polymer backbone, as supported by SAXS data presented in Supplementary Fig. 24.

S5.1 First Passage Time (FPT) distribution for ion mobility

In Fig. 3h, we use the FPT distribution to illustrate changes in ion mobility across two distinct RH regimes. It is important to note that some molecules may frequently enter and exit the boundary during the observed period. To ensure that this event does not affect the FPT distribution, we have calculated the FPT distribution using different sampling intervals ranging from 10 ps to 500 ps, to cover a wide time range of ion transport events. Fig. 39a-b show that the distribution curves overlap and display no significant changes when changing sampling intervals. This also

indicates that, for both low (Fig. 39a) and high RH (Fig. 39b), the choice of sampling interval does not affect the FPT distribution trend in our calculations.

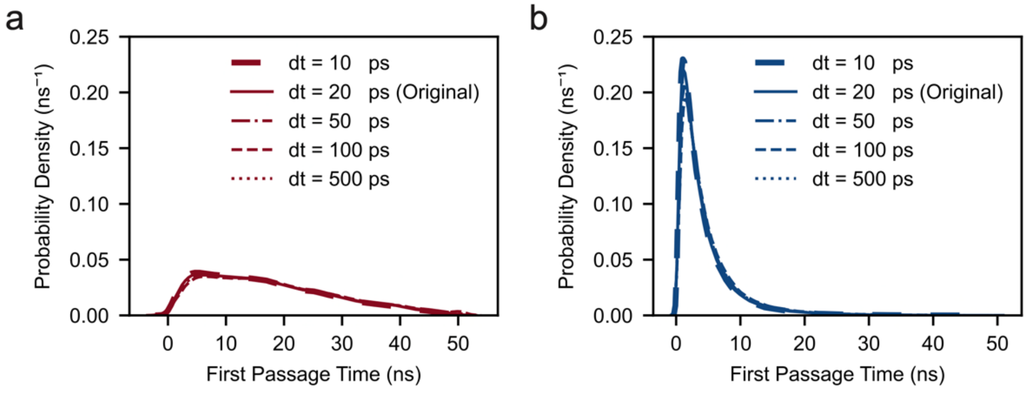

Supplementary Fig. 39 | First Passage Time (FPT) distribution for ion mobility at different sampling intervals. FPT distributions are calculated with sampling intervals ranging from dt = 10 ps to 100 ps at **a**, 25% and **b**, 85% RH. The curves for different sampling intervals overlap closely at both low and high hydration level, indicating that the sampling interval has minimal effect on the FPT distribution. The sampling interval of 20 ps is the original interval used to calculate the FPT distribution in Fig. 3h.

The minimal effect observed in the FPT distribution across different sampling intervals can be attributed to the dominance of long-range ion transport dynamics over short-range, transient boundary crossings. This confirms that the choice of 1 nm as the distance for measuring FPT is sensible, as it represents a long-range transport event compared to boundary crossings. Ions frequently entering and exiting the boundary undergo rapid, local movements that do not significantly impact the overall FPT. These local movements are averaged out in the measurement, which focuses on the time required for an ion to traverse 1 nm. The consistency in the FPT distribution across various sampling intervals ensures the robustness of our results. Additionally, it holds for both low (25% RH) and high (85% RH) hydration levels, further confirm that this effect is negligible.

Supplementary references

1. Chen, C., Tse, Y. L. S., Lindberg, G. E., Knight, C. & Voth, G. A. Hydroxide Solvation and Transport in Anion Exchange Membranes. *J Am Chem Soc* **138**, 991–1000 (2016).
2. Tuckerman, M. E., Marx, D. & Parrinello, M. The nature and transport mechanism of hydrated hydroxide ions in aqueous solution. *Nature 2002 417:6892* **417**, 925–929 (2002).
3. Salvatore, D. A. *et al.* Designing anion exchange membranes for CO2 electrolysers. *Nature Energy 2021 6:4* **6**, 339–348 (2021).
4. Chu, W. *et al.* Understanding Ion Mobility in P2VP/NMP+I- Polymer Electrolytes: A Combined Simulation and Experimental Study. *Macromolecules* (2020)
5. Webb, M. A. *et al.* Systematic computational and experimental investigation of lithium-ion transport mechanisms in polyester-based polymer electrolytes. *ACS Cent Sci* **1**, 198–205 (2015).
6. Bennington, P. *et al.* Role of solvation site segmental dynamics on ion transport in ethylene-oxide based side-chain polymer electrolytes. *J Mater Chem A Mater* **9**, 9937–9951 (2021).
7. Sharon, D. *et al.* Intrinsic Ion Transport Properties of Block Copolymer Electrolytes. *ACS Nano* **14**, 8902–8914 (2020).
8. Sharon, D. *et al.* Molecular Level Differences in Ionic Solvation and Transport Behavior in Ethylene Oxide-Based Homopolymer and Block Copolymer Electrolytes. *J Am Chem Soc* **143**, 3180–3190 (2021).
9. Roberts, S. T., Ramasesha, K., Petersen, P. B., Mandal, A. & Tokmakoff, A. Proton transfer in concentrated aqueous hydroxide visualized using ultrafast infrared spectroscopy. *Journal of Physical Chemistry A* **115**, 3957–3972 (2011).
10. Roberts, S. T., Mandal, A. & Tokmakoff, A. Local and collective reaction coordinates in the transport of the aqueous hydroxide ion. *Journal of Physical Chemistry B* **118**, 8062–8069 (2014).
11. Roberts, S. T. *et al.* Observation of a Zundel-like transition state during proton transfer in aqueous hydroxide solutions. *Proc Natl Acad Sci U S A* **106**, 15154–15159 (2009).
12. Detallante, V. *et al.* Water vapor sorption in naphthalenic sulfonated polyimide membranes. *J Memb Sci* **190**, 227–241 (2001).
13. Mecheri, B. *et al.* DSC and DVS investigation of water mobility in Nafion/zeolite composite membranes for fuel cell applications. *Journal of Physical Chemistry C* **116**, 20820–20829 (2012).
14. Zheng, Y. *et al.* Water Uptake Study of Anion Exchange Membranes. *Macromolecules* **51**, 3264–3278 (2018).

15. Adamski, M., Peressin, N. & Holdcroft, S. On the evolution of sulfonated polyphenylenes as proton exchange membranes for fuel cells. *Materials Advances* **2**, 4966–5005 (2021).

16. Siu, A., Schmeisser, J. & Holdcroft, S. Effect of Water on the Low Temperature Conductivity of Polymer Electrolytes. *Journal of Physical Chemistry B* **110**, 6072–6080 (2006).

17. Canongia Lopes, J. N., Deschamps, J. & Pádua, A. A. H. Modeling Ionic Liquids Using a Systematic All-Atom Force Field. *Journal of Physical Chemistry B* **108**, 2038–2047 (2004).

18. Leontyev, I. V. & Stuchebrukhov, A. A. Electronic continuum model for molecular dynamics simulations of biological molecules. *J Chem Theory Comput* **6**, 1498–1508 (2010).

19. Fong, K. D. *et al.* Ion Transport and the True Transference Number in Nonaqueous Polyelectrolyte Solutions for Lithium Ion Batteries. *ACS Cent Sci* **5**, 1250–1260 (2019).

20. Dubey, V., Maiti, A., & Daschakraborty, S. Predicting the solvation structure and vehicular diffusion of hydroxide ion in an anion exchange membrane using nonreactive molecular dynamics simulation. *Chemical Physics Letters* **755**, 137802 (2020).
